# Influence of mental stress and environmental toxins on circadian clocks: Implications for redox regulation of the heart and cardioprotection

**DOI:** 10.1111/bph.14949

**Published:** 2020-02-04

**Authors:** Huige Li, Aoife B. Kilgallen, Thomas Münzel, Eva Wolf, Sandrine Lecour, Rainer Schulz, Andreas Daiber, Linda W. Van Laake

**Affiliations:** ^1^ Department of Pharmacology Medical Center of the Johannes Gutenberg University Mainz Germany; ^2^ Division Heart and Lungs and Regenerative Medicine Centre University Medical Centre Utrecht and Utrecht University Utrecht Netherlands; ^3^ Center of Cardiology 1, Molecular Cardiology Medical Center of the Johannes Gutenberg University Mainz Germany; ^4^ German Center for Cardiovascular Research (DZHK), Partner Site Rhine‐Main Mainz Germany; ^5^ Structural Chronobiology, Institute of Molecular Physiology Johannes Gutenberg University Mainz Germany; ^6^ Structural Chronobiology Institute of Molecular Biology Mainz Germany; ^7^ Hatter Institute for Cardiovascular Research in Africa University of Cape Town Cape Town South Africa; ^8^ Institute for Physiology Justus‐Liebig University Giessen Giessen Germany

## Abstract

Risk factors in the environment such as air pollution and mental stress contribute to the development of chronic non‐communicable disease. Air pollution was identified as the leading health risk factor in the physical environment, followed by water pollution, soil pollution/heavy metals/chemicals and occupational exposures, however neglecting the non‐chemical environmental health risk factors (e.g. mental stress and noise). Epidemiological data suggest that environmental risk factors are associated with higher risk for cardiovascular, metabolic and mental diseases, including hypertension, heart failure, myocardial infarction, diabetes, arrhythmia, stroke, depression and anxiety disorders. We provide an overview on the impact of the external exposome comprising risk factors/exposures on cardiovascular health with a focus on dysregulation of stress hormones, mitochondrial function, redox balance and inflammation with special emphasis on the circadian clock. Finally, we assess the impact of circadian clock dysregulation on cardiovascular health and the potential of environment‐specific preventive strategies or “chrono” therapy for cardioprotection.

**LINKED ARTICLES:**

This article is part of a themed issue on Risk factors, comorbidities, and comedications in cardioprotection. To view the other articles in this section visit http://onlinelibrary.wiley.com/doi/10.1111/bph.v177.23/issuetoc

**Abbreviations**
AMI: acute myocardial infarction
BMAL1: brain and muscle arnt‐like protein‐1
CLOCK: circadian locomotor output cycles protein kaput
CRY: cryptochrome
dCRY: *Drosophila* cryptochrome
eNOS: endothelial NOS
FBXL3: F‐box/leucine rich‐repeat protein 3
FOXO: forkhead box O
HPA: hypothalamic–pituitary–adrenal
LCPS: lifelong chronic psychosocial stress
mCRY1: mammalian cryptochrome 1
MI: myocardial infarction
mPER2: mammalian period 2
mPER2‐M: messenger period 2‐mouse
NASH: non‐alcoholic steatohepatitis
NPAS2: neuronal PAS domain‐containing protein 2
NRF2: nuclear factor erythroid 2‐related factor
PM2.5: particulate matter 2.5
ROFA: residual oil fly ash
ROR‐α: RAR‐related orphan receptor
SCN: superchiasmatic nucleus
SIRT1: sirtuin 1
VSMCs: vascular smooth muscle cells
WT: wild type
ZT: Zeitgeber time

## 1 INTRODUCTION

### 1.1 Environmental risk factors, disease burden and global mortality

The global burden of disease has shifted from communicable, maternal, perinatal and nutritional causes to non‐communicable diseases such as atherosclerosis. Important novel factors in the physical environment may facilitate the development of chronic non‐communicable disease (Landrigan et al., 2018). With industrialisation and globalisation, the importance of new environmental risk factors such as air pollution (Lelieveld et al., 2019; Munzel et al., 2018) is becoming increasingly significant. In particular, traffic noise (from road, aircraft and railway) is a potential novel cardiovascular risk factor (Kempen, Casas, Pershagen, & Foraster, 2018) and a large body of evidence indicates that noise can cause cardiovascular, metabolic and mental disease, including hypertension, heart failure, myocardial infarction (MI), diabetes, arrhythmia, stroke, depression and anxiety disorders, observations that are mainly based on not only association studies but also controlled interventional studies (Munzel et al., 2018). Robust epidemiological evidence for detrimental health effects of environmental risk factors and mechanistic insights into the underlying pathophysiology are mostly based on association studies (with only few controlled interventional studies) and exist for the environmental risk factors mental stress (e.g. social isolation and work stress), air pollution (e.g. particulate matter), noise exposure (e.g. traffic/occupational sources) and chemical pollution (e.g. heavy metals and pesticides; for references, see Supporting Information). Diseases caused by pollution were responsible for an estimated 9 million premature deaths in 2015—16% of all deaths worldwide—three times more deaths than from acquired immunodeficiency disorder syndrome, tuberculosis and malaria combined, and 15 times more than from all wars and other forms of violence (Landrigan et al., 2018). In the most severely affected countries, pollution‐related disease is responsible for more than one death in four (Landrigan et al., 2018). However, it is important to keep in mind that there are significant differences in the regional distribution and accordingly clinical relevance of these environmental stressors (e.g. sub‐Saharan region has different water purity and sanitation than Western European countries; Landrigan et al., 2018). The same applies for the source and characteristics (severe life stress vs. work strain) of mental stress. More epidemiological background information on environmental risk factors and health effects can be found in the Supporting Information.

Current concepts propose that most of these environmental risk factors lead to chronic stress reactions with increased stress hormones (cortisol, adrenaline, and noradrenaline), oxidative stress, and activation of inflammatory pathways leading to pathophysiologic alterations that contribute directly or indirectly to the initiation and progression of cardiovascular disease (Munzel & Daiber, 2018). Therefore, a significant part of the present review is dedicated to mental stress exposures and stress response pathways, as previously highlighted by us (Daiber et al., 2019; Xia & Li, 2018), but here with a strong focus on circadian clock dysregulation as a potential down‐stream pathomechanism of environmental risk factors. Circadian clock dysregulation by environmentally triggered adverse redox regulation/oxidative stress and its impact on cardiovascular damage will be discussed in detail.

### 1.2 The exposome as the totality of all environmental exposures

**FIGURE 1:**
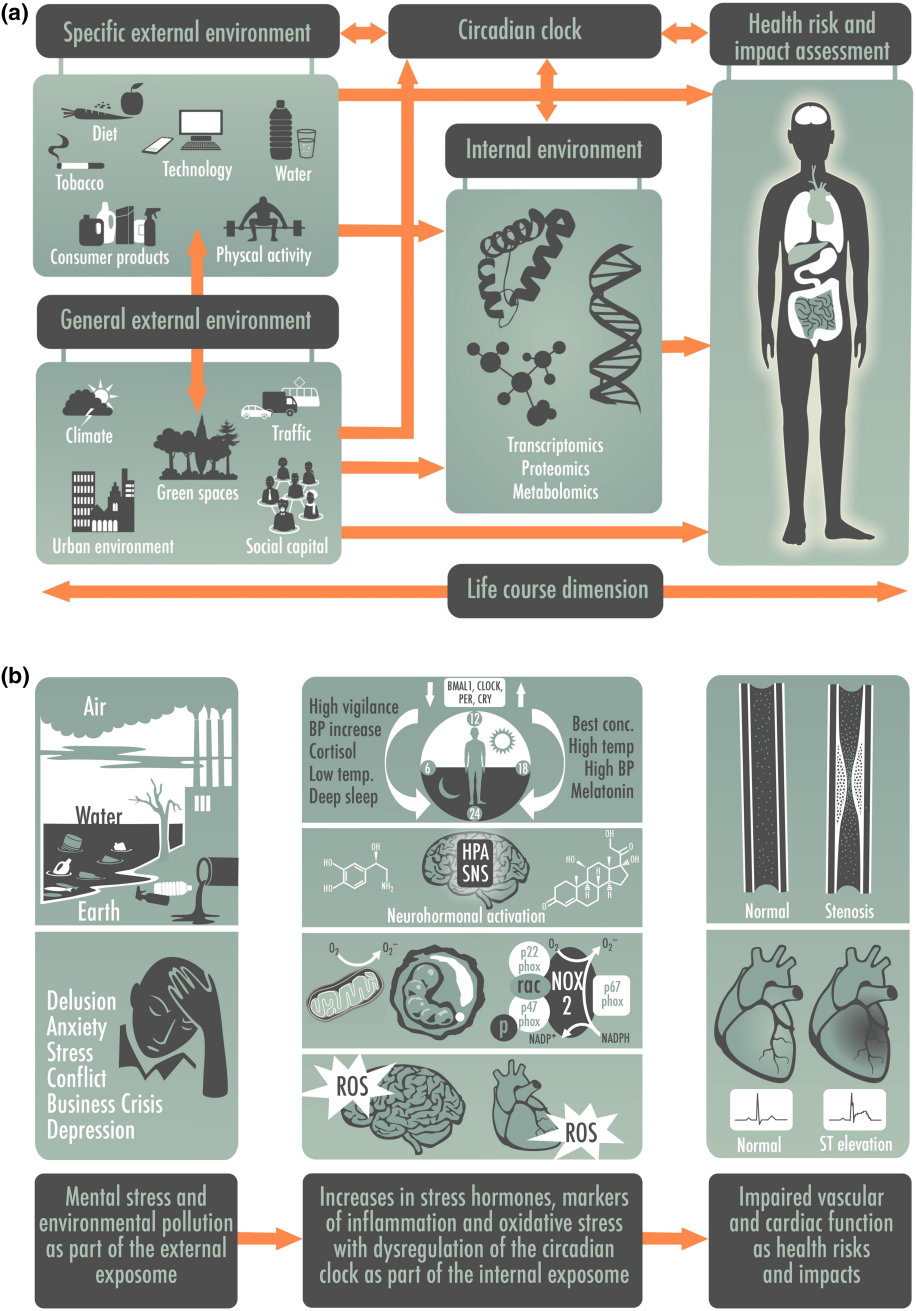
(a) The exposome concept. The external exposome represents the sum of all environmental exposures including behavioural/lifestyle factors, whereas the internal exposome reflects all biological changes (viz. expression levels of biomolecules characterised by the transcriptome and epigenome, proteome, metabolome and lipidome) that are triggered by environmental factors. Exposome research is dedicated to the identification of associations between environmental exposures and health effects (e.g. disease and mortality). Redrawn from Vrijheid (2014) with permission (Copyright © BMJ Publishing Group Ltd & British Thoracic Society. All rights reserved) and modified from Daiber et al. (2019) with permission (© 2019 International Union of Biochemistry and Molecular Biology). (b) The external exposome (e.g. mental stress and environmental pollution) confer changes of the internal exposome (e.g. altered circadian clock by forward/backward shift, stress hormones, inflammation and oxidative stress) leading to health risks and disease conditions (e.g. atherosclerosis, vascular stenosis and myocardial infarction)

The sum of all contributions of the environment to human physiology and pathophysiology between conception and death is reflected by the exposome, which was described in detail by Christopher P. Wild in 2005. In addition to the above‐mentioned environmental stressors (e.g. different “pollutomes”), also behavioural or lifestyle (e.g. diet, smoking and physical activity) and more general environmental factors (e.g. socio‐economic status, urban environment, pathogens, UV radiation and climate) define the individual exposome and are subcategorised into the specific and general external environment (Figure 1a; Sainani, 2016; Vrijheid, 2014). What is usually measured by exposome studies is the internal environment, the transcriptome (including the epigenome), proteome and metabolome but also the microbiome and correlations are made with the environmental factors as well as the health outcomes, allowing an estimation of the contribution of specific or general environmental factors to health risks, disease burden and mortality. The knowledge of the internal environment provides deep mechanistic insights on these associations and allows interactome and diseasome bioinformatical mapping to gain new knowledge on environment‐triggered disease and potential overlap with classical metabolic, cardiovascular, or neurodegenerative diseases as well as cancer. Importantly, the influence of the exposome on development and progression of chronic diseases may even exceed those relating to genetic predisposition (Rappaport, 2016).

Here, we will discuss in detail the influence of the external exposome by exposure to the non‐chemical environmental risk factor mental stress (e.g. by work strain, social isolation, sleep deprivation, and noise exposure) as well as the chemical factors air/water/soil pollution (e.g. by particulate matter, heavy metals and pesticides) on changes of the internal exposome (Figure 1b). These changes of the internal exposome will be illustrated by modulation of stress hormone levels, oxidative stress markers, and inflammatory pathways with emphasis on changes in circadian clock pathways and their interaction with redox regulatory and inflammatory pathways. Thereby, we will establish a connection of the external exposome with circadian clock dysregulation. Finally, we will provide a link between the external exposome (environmental exposures) via changes of the internal exposome, namely, stress responses and circadian clock dysregulation and health impact/risk in the form of cardiovascular complications. The role of a synchronised or dysregulated circadian clock for cardiovascular health and disease will also be addressed in full detail. The circadian clock plays a special role since it can also crosstalk with the specific external environment, which means it can directly modulate our behaviour with regard to diet, activity, sleep, cognitive functions and others.

## 2 OXIDATIVE STRESS, REDOX REGULATION, AND CIRCADIAN CLOCK

Oxidative stress is a common feature of all major environmental exposures (Munzel & Daiber, 2018) and is well documented for mental stress (Xia & Li, 2018), traffic noise (Munzel et al., 2017), air pollution (Munzel, Gori, et al., 2018), heavy metal (Cosselman, Navas‐Acien, & Kaufman, 2015) or pesticide exposures (Drechsel & Patel, 2008; Miguel et al., 2018) and, of course, for life style risk factors such as tobacco/e‐cigarette/water‐pipe smoking (Golbidi, Li, & Laher, 2018). The sources of ROS formation in response to environmental triggers comprise NADPH oxidases, mitochondrial respiratory complexes and an uncoupled endothelial NOS (eNOS). Here, we discuss the potential interaction between oxidative stress pathways and circadian clock dysregulation in response to environmental exposures. Almost all studies deal with mammalian (mostly mouse) circadian clock besides the few clearly defined studies in *Drosophila* flies.

### 2.1 Circadian rhythms

Circadian rhythms can be defined as biological rhythms that occur over a 24‐hr period, allowing living organisms to synchronise their physiological processes and adapt to environmental fluctuations that occur throughout the day and night (Crnko, Du Pre, Sluijter, & Van Laake, 2019). Diurnal rhythms differ to circadian rhythms because they are patterns that occur over 24 hr in conditions with input from the environment, whereas circadian rhythms can be self‐sustaining even without external cues (Reppert & Weaver, 2002). In practice, these terms are often used interchangeably. These circadian rhythms are controlled by the master clock, consisting of 20,000 neurons in the suprachiasmatic nucleus (SCN) of the hypothalamus and peripheral clocks, which are found in almost every mammalian tissue (Buhr & Takahashi, 2013). Light, the main external stimulus, known as a Zeitgeber or time‐giver (ZT), is received by the photoreceptors in the retina and is directly transmitted to the SCN of the hypothalamus (Du Pre et al., 2014) and synchronises the peripheral clocks through the release of neural and humoral signals such as melatonin (Cajochen, Krauchi, & Wirz‐Justice, 2003). Besides input from the master clock, peripheral clocks respond to food, exercise and social cues.

Peripheral clocks are endogenous and autonomous complex molecular rhythms generated by tightly controlled transcriptional–translational feedback loops that cycle every 24 hr (Merbitz‐Zahradnik & Wolf, 2015; Reppert & Weaver, 2002), which basically also accounts for the molecular clock (Mohawk, Green, & Takahashi, 2012). In mammals, these loops are generated by clock core genes such as circadian locomotor output cycles protein kaput (*Clock*), period 1, 2 and 3 (*Per1*, *Per2*, and *Per3*), cryptochrome 1 and 2 (*Cry1* and *CRY2*) and brain and muscle arnt‐like protein‐1 (*Bmal1* and *Bmal2*; also described in section “6.2 Circadian rhythms in cardiovascular pathophysiology” and figure therein). Clock and Bmal1 (or its paralog NPAS2), act as positive regulators, heterodimerise and bind to the E box promotors of Per and Cry to initiate their gene transcription, creating a positive feedback loop (Merbitz‐Zahradnik & Wolf, 2015; Reppert & Weaver, 2002). Translated Per and Cry act as a negative feedback loop by inhibiting the transactivation of *Bmal1* and *Clock* and, in doing so, turn off their own expression. This feedback loop is interconnected with the rhythmic expression of *Rev‐erbα* and *Rorα*, which controls the expression of *Bmal1* (Merbitz‐Zahradnik & Wolf, 2015; Mohawk et al., 2012).

### 2.2 Redox regulatory mechanism in circadian clock

**FIGURE 2:**
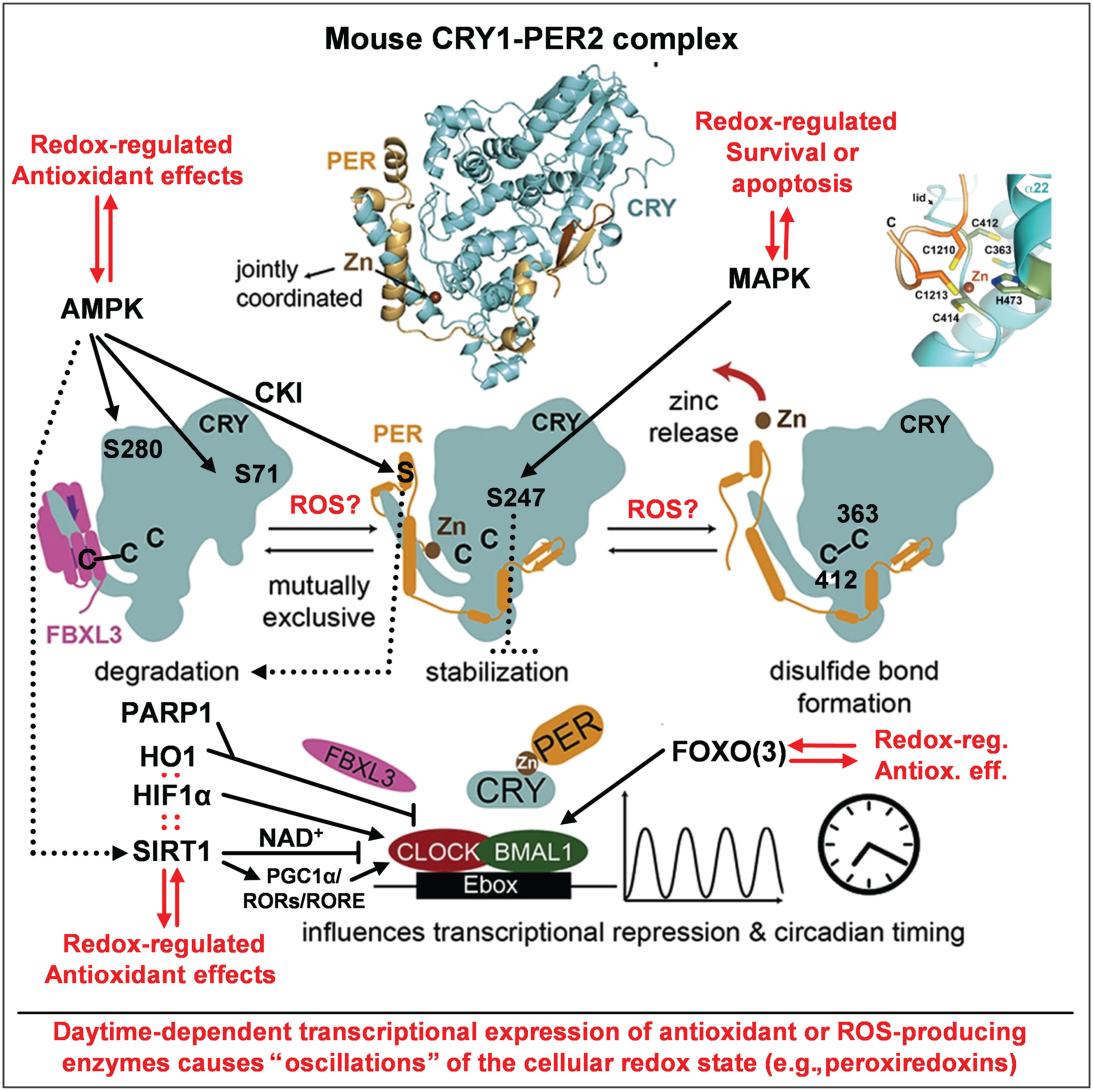
Proposed mechanisms of redox regulation of the circadian clock. A number of redox‐sensitive cysteine thiol groups (C363 and C412) and a zinc‐sulfur centre (C1210 and C1213 of PER2, C414 and H473 of CRY1, top right) were identified in mammalian CRY that act as redox switches via disulphide bond formation controlling PER binding and thereby the activity of the CLOCK/BMAL1 complex. The scheme also contains other redox‐sensitive pathways in the regulation of circadian rhythm such as redox‐sensitive kinases AMPK or MAPK. AMPK phosphorylates S71 and S280 to affect the affinity of CRY1 for the E3 ligase FBXL3 and thereby CRY stability. The MAPK phosphorylates S247 to affect CRY‐dependent transcriptional repression of BMAL1/CLOCK. Furthermore, stress response proteins such as PARP‐1, HO‐1, HIF‐1α, PGC‐1α, FOXO3, and the histone deacetylase SIRT‐1 affect the circadian clock by modifying the transcriptional activity of BMAL1/CLOCK (see main text and extended information in 2.2 in the Supporting Information for details). Vice versa, the expression of several antioxidant and ROS‐producing enzymes is controlled by the circadian clock and thereby contributes to cellular redox homeostasis. Modified from graphical abstract in Schmalen et al. (2014) with permission (Copyright © 2014 Elsevier Inc. All rights reserved)

Crystal structures of the mammalian/mouse and *Drosophila* cryptochromes (mCRY1 and dCRY) provided valuable insights into redox regulation of the circadian clock (Czarna et al., 2013). While dCRY is a blue‐light photoreceptor mediating light‐synchronisation/entrainment of the *Drosophila* circadian clock (see Supporting Information), the two mammalian cryptochrome homologues, mCRY1 and mCRY2, are integral clock components that represses mCLOCK/mBMAL1‐dependent transcription in a circadian manner. The transcriptional repressor activity and stability of mCRY1 and mCRY2 is critically regulated by their tight interaction with the clock proteins mPER1, mPER2 or the E3‐ligase component FBXL3 (reviewed in Merbitz‐Zahradnik & Wolf, 2015). The crystal structure of mouse CRY1 (mCRY1) visualised AMPK phosphorylation sites at Ser71 and Ser280 that regulate mCRY1 stability in response to the cellular metabolic as well as redox state (Figure 2; Czarna et al., 2013). Of note, AMPK represents a key stress response protein with antioxidant properties (Schulz et al., 2008), and AMPK itself is redox regulated (Shirwany & Zou, 2014). More details on redox regulation of CRY and its interaction partners can be found in the Supporting Information.

The crystal structure of the mouse mCRY1/mPER2 complex, which is essential for the negative regulation of mCLOCK/BMAL1‐dependent transactivation of clock‐ and clock‐controlled genes (Schmalen et al., 2014), unexpectedly revealed that Cys1210/Cys1213 residues in mPER2 and Cys414/His473 residues in mCRY1, form a tetrahedral zinc‐complex (termed “zinc interface”) stabilising the mCRY1/mPER2 complex (Figure 2, closeup view upper right; Schmalen et al., 2014). Interestingly, the Cys412‐Cys363 disulphide bond observed in mCRY1 is absent in the CRY1‐PER2 complex. Here, the jointly co‐ordinated zinc ion prevents oxidation/disulphide bond formation of the CRY1 cysteine residues, which likely enhances the flexibility of the PER2‐interacting loop to facilitate mPER2 binding (Schmalen et al., 2014). These mechanistic insights support not only a regulation of mCRY1 and the mCRY1/mPER2 complex (and thereby transcriptional repression of clock‐regulated genes) by the cellular redox state, which would impact mCRY1 cysteine oxidation, but also zinc incorporation in the mCRY1/PER2 complex by changing the intracellular pool of “free” zinc ions (Oteiza, 2012). Thereby, these findings provide a feasible mechanism how oxidative stress may cause a dysregulation of the circadian clock and accordingly cause large (adverse) changes in clock‐regulated genes in response to environmental and classical risk factors. Interestingly, mice overexpressing a zinc binding deficient mCRY1(C414A) mutant protein showed a long 28‐hr circadian period, abnormal entrainment behaviour as well as symptoms of diabetes, including reduced cell proliferation and insulin secretion (hypoinsulinaemia; Okano, 2016), clearly demonstrating the functional importance of zinc‐dependent mCRY1/mPER2 complex formation for circadian dynamics and metabolic regulation. Overall, the structural analyses of mouse cryptochromes suggest an important role of cysteine redox modifications and of a zinc‐sulphur complex for the general regulation of the circadian clock‐dependent gene expression by the temporal redox oscillations observed in most tissues and in particular the redox regulation of hypo‐ and hyperinsulinaemia with development of diabetes. Other redox regulatory mechanisms in the circadian clock are based on AMPK/SIRT1, PARP1, FOXO3, HIF‐1α, or haem oxygenase‐1 (mostly reviewed in Reinke & Asher, 2019, and summarised in Figure 2) and are described in detail in the Supporting Information.

### 2.3 Association of circadian clock with cellular redox state in health and disease

Likewise, the circadian clock itself can cause redox oscillations by daytime‐dependent transcriptional expression of antioxidant genes or ROS‐producing genes. Examples are the circadian control of NRF2 activation by brain‐derived neurotrophic factor (BDNF), the major neurotrophin expressed in adult in rodent brain with a key role in neuronal survival, differentiation and synaptic plasticity (Ishii, Warabi, & Mann, 2019). BDNF‐dependent activation of NRF2 in astrocytes is mediated by receptor combination of the truncated form of TrkB.T1 and the low affinity receptor p75^NTR^, where the latter is fully controlled by the activity of the CLOCK/BMAL1 complex. NRF2‐dependent expression of antioxidant genes in astrocytes is obviously essential for protection of dopaminergic neurons from ferroptosis. Neuroprotection is also controlled by circadian clock via regulation of intracellular GSH levels, which represents a major antioxidant in neuronal cells (Kinoshita, Aoyama, & Nakaki, 2018). The circadian clock itself is regulated by a network of microRNAs that are also implicated in the regulation of important antioxidant enzymes (e.g. SODs, catalase, and GPx/Prx/Trx isoforms) and enzymes involved in GSH synthesis or reduction. The circadian control of antioxidant and/or ROS‐producing gene expression can be easily seen by daytime‐dependent “redox oscillations” envisaged by the rhythmic changes of peroxiredoxin (Prx) redox state (e.g. reduced thiols, disulphide, sulfenic, sulfinic and sulfonic acid content; Rey & Reddy, 2015; Figure 2).

Vice versa, the reciprocal regulation between the cellular redox state and the circadian clock has been proposed and termed “redox control of cellular timekeeping” (Putker & O'Neill, 2016), which is in good accordance with the above provided insights in redox regulatory mechanism within the circadian clock. It was proposed that metabolic regulation is not simply an output function of the circadian clock, but nutrient, energy, and cellular redox state signal back to cellular clocks in order to reinforce circadian rhythmicity and to adapt physiology to temporal tissue‐specific needs (Reinke & Asher, 2019). Accordingly, circadian clock is frequently dysregulated under oxidative stress conditions, and some examples are briefly discussed here. Redox signalling is important for vascular smooth muscle contraction and the producing/degrading enzymes of ROS or NO are under circadian control, which has been proven by endothelial/vascular dysfunction and oxidative stress in animals (mostly mice) with dysregulated circadian clock by genetic deletion of circadian key regulators (Anea et al., 2012; Crnko, Cour, Van Laake, & Lecour, 2018; Viswambharan et al., 2007). Circadian clock genes (*Clock*, *Bmal1*, *Cry2*, and *Per2*) were dysregulated in mice with high fat diet induced non‐alcoholic steatohepatitis (NASH), affecting clock‐regulated lipid metabolism proteins (Rev‐Erbα, RORα and SREBP1c) leading to further impairment of lipid metabolism and fatty liver development (Bruce et al., 2016). Most likely, circadian clock dysregulation in the NASH model was triggered by altered redox balance and impaired sirtuin (SIRT1 and SIRT3) activity. More examples can be found in the Supporting Information.

## 3 MENTAL STRESS EFFECTS ON OXIDATIVE STRESS, CIRCADIAN CLOCK AND CARDIOVASCULAR HEALTH/DISEASE

According to the concept of Hans Selye, the stress reaction leading to disease can be separated as follows (Selye, 1955): “The three stages of the stress syndrome are (i) the alarm reaction, in which adaptation has not yet been acquired; (ii) the stage of resistance, in which adaptation is optimum and (iii) the stage of exhaustion, in which the acquired adaptation is lost again.” A detailed description of the underlying biochemical processes and a guideline on how to measure stress conditions are described in this pioneering work.

### 3.1 Mental stress effects on oxidative stress and cardiovascular health/disease

**FIGURE 3:**
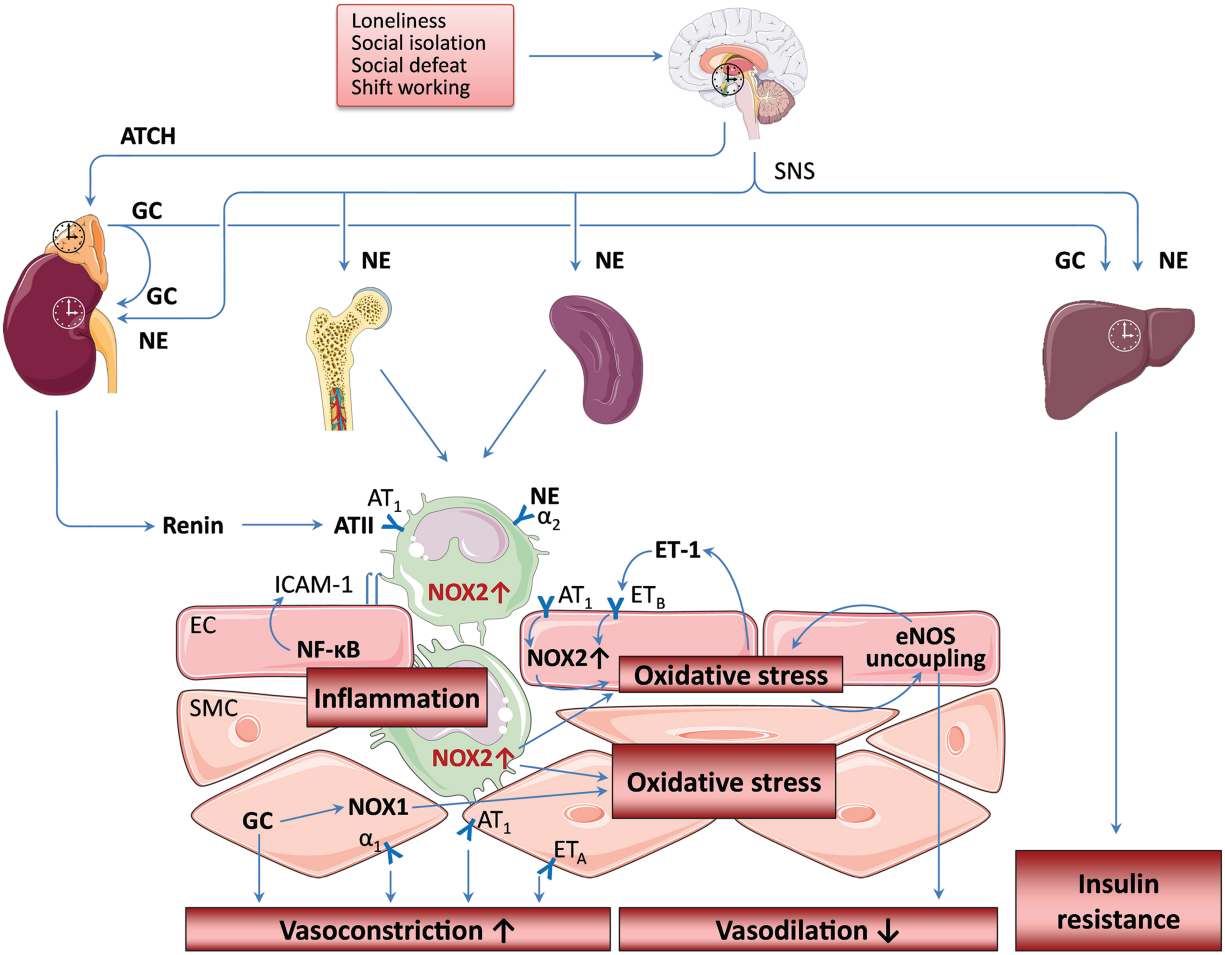
Mental stress‐induced circadian dysregulation. Loneliness, social isolation, social defeat and shift working stress lead to activation of the hypothalamic–pituitary–adrenal (HPA) axis and the sympathetic nervous system (SNS) and induce changes in circadian clocks. SNS activation enhances monocytopoiesis in the bone marrow resulting in expansion of immature proinflammatory monocytes. In addition, SNS also stimulates monocyte egress from the spleen. SNS stimulates renin secretion and the production of angiotensin II (ATII). ATII activates NADPH oxidase (NOX2) in endothelial cells (EC) causing oxidative stress, which may induce uncoupling of the endothelial NOS (eNOS) through tetrahydrobiopterin oxidation and eNOS S‐glutathionylation. The uncoupling of eNOS not only reduces NO production and vasodilation but also potentiates the pre‐existing oxidative stress. The SNS neurotransmitter noradrenaline (NE) enhances NOX expression and promotes adhesion of immune cells to the vascular wall. Infiltrating immune cells (e.g. neutrophils and monocytes/macrophages) causes vascular oxidative stress via NOX2 activity, which is enhanced by ATII via the AT_1_ receptor. Oxidative stress enhances endothelin‐1 (ET‐1) production, which, in turn, stimulates NOX2‐mediated superoxide production, resulting in a vicious cycle. Oxidative stress in ECs also leads to activation of NF‐κB and the induction of adhesion molecules resulting in vascular inflammation. Glucocorticoids (GC) enhances NOX1 expression in vascular smooth muscle cells (SMC) and potentiates vasoconstriction by induced NE, ET‐1, or ATII. The images of brain, bone, spleen and monocytes used in this figure are from Servier Medical Art licensed under the Creative Commons Attribution 3.0 Unported License. The scheme is partly adopted from our previous publications: Xia et al. (2018) and Golbidi et al. (2018) with permission (Copyright © 2018, Mary Ann Liebert, Inc.)

Chronic mental stress has been associated with cardiovascular risk factors such as increased BP and dyslipidaemia, increased blood viscosity and blood glucose, in addition in humans activation of blood clotting factors (Babisch, 2002). Animal models revealed that when mice were subjected to daily restraint stress and cage‐switch stress for 1 week, they displayed significant activation of the immune system and development of hypertension (Marvar & Harrison, 2012) by mechanisms explained in Figure 3. Findings from a large pan‐European epidemiological study in 124,808 diabetes‐free subjects indicate that job strain is a risk factor for type 2 diabetes in men and women independent of other lifestyle factors such as obesity and physical inactivity (Nyberg et al., 2014). A meta‐analysis of nine case–control and cohort studies of good methodological quality showed positive associations between hypertension and job strain (Babu et al., 2014). Another meta‐analysis suggested that a greater responsiveness to acute mental stress has an adverse effect on future cardiovascular risk status (including elevated BP, hypertension, left ventricular mass, subclinical atherosclerosis and clinical cardiac events), supporting the use of methods of managing stress responses to improve cardiovascular prognosis (Chida & Steptoe, 2010). A meta‐analysis of 196,380 males and females from 14 European cohort studies revealed that the association of job strain with ischaemic stroke was even observed after further adjustment for socio‐economic status (Fransson et al., 2015). Finally, a meta‐analysis of 10 prospective cohort and four case–control design studies revealed that exposure to psychosocial stress (general or work stress or stressful life events) is independently associated with increased risk of stroke (Booth et al., 2015).

In addition, growing evidence indicates that the peripheral circadian clock plays a role in regulating vascular redox status and endothelial function in mice (Patrick & Harrison, 2018). For instance, the levels of tetrahydrobiopterin, an essential cofactor for eNOS, and key enzymes that regulate biopterin bioavailability exhibit a circadian expression pattern (Anea et al., 2012). Bmal1 deficiency in mice leads to reduced tetrahydrobiopterin levels, eNOS uncoupling, and endothelial dysfunction (Anea et al., 2012). More examples can be found in the Supporting Information. The impact of mental stress on oxidative stress pathways and inflammation was reviewed in full detail by Siegrist and Sies (2017) as well as in two recent articles within the forum issue “Oxidative stress and redox signaling induced by the environmental risk factors mental stress, noise and air pollution” (Xia & Li, 2018) by mechanisms explained in (Figure 3).

### 3.2 Mental stress effects on circadian clock

The nature of the stress response is dependent on the type of the stressor. Internal stressors (e.g. oxidative stress, hypoglycaemia and haemorrhage) induce higher increase in circulating glucocorticoids (GCs) at the animal's activity phase. In contrast, external stressors (e.g. restraint/immobilisation, foot shock, tail shock, and shaking) usually lead to a higher adrenocorticotropic hormone (ACTH)/GC release and BP elevation during the animal's inactive phase (Helfrich‐Forster, 2017). A variety of stressor types, including restraint stress, social defeat stress, or placement on an elevated platform, food or water deprivation, continuous lightning, cage tilts and soiled cages, have been shown to entrain (peripheral) clocks (Helfrich‐Forster, 2017; Koch, Leinweber, Drengberg, Blaum, & Oster, 2017; Tahara et al., 2015). Mental stress in modern human societies, however, is mainly caused by social interactions (Koch et al., 2017). Therefore, we focus in the present review article on two types of psychosocial stressors, that is, the social defeat stress and social isolation. Moreover, we will also address the effect of noise on circadian rhythm, because the level of ambient noise in our environment has been continuously growing with the increasing urbanisation.

### 3.3 Social defeat stress and circadian clocks

The social defeat stress is a common rodent model for social stress with a high translational relevance (Koch, Leinweber, et al., 2017). In this model, the experimental mouse (intruder) is placed for a specific time period into the cage of an aggressive resident (the resident‐intruder paradigm), resulting in a subordination mimicking social conflicts in humans (Koch, Leinweber, et al., 2017).

The peripheral clocks are much more sensitive to social defeat stress than the SCN clock and can be modulated even by an acute social defeat stress. A sub‐acute social defeat stress (2‐hr daily at ZT4‐6 over three consecutive days) caused clear phase advance of bioluminescence rhythms in the kidneys, livers and submandibular glands of the intruder mice (Tahara et al., 2015). A single social defeat stress (at ZT3‐3.5) was sufficient to phase advance the clock in the adrenal gland, although it failed to shift the clock in the pituitary as chronic stress did, indicating that the adrenal clock is more sensitive to social stress (Razzoli, Karsten, Yoder, Bartolomucci, & Engeland, 2014).

In the chronic social defeat stress experiment done by Razzoli et al., stable resident/intruder pairs were established. During the 2‐week stress phase, the two male mice were allowed to freely interact for a maximum of 10 min at ZT3‐3.5 each day. After the 10‐min interaction, the two animals remained in the same cage but were separated by a perforated partition, which allowed continuous visual, auditory, and olfactory contact but no physical interaction throughout the remainder of the 24 hr (Razzoli et al., 2014). Chronic social defeat stress for 2 weeks led to hyperphagia, body weight gain and overall increased feed conversion efficiency. It phase advanced the adrenal (~2 hr) and the pituitary (~1 hr) PER2 rhythm in the stressed mice compared with control mice. The phase shift occurred in the absence of changes in the period of the PER2 rhythms, indicating that adrenal and pituitary clocks were reset by the chronic social defeat stress (Razzoli et al., 2014). More examples can be found in the Supporting Information.

In a recently developed model of lifelong chronic psychosocial stress (LCPS) in male mice, a causal role for social defeat stress on shortening lifespan and increasing cardiovascular disease risk has been demonstrated (Razzoli et al., 2018). The LCPS model consists of a baseline phase of 5 days, followed by a 4‐week psychosocial stress phase and an aging phase until death. During the baseline phase, all mice are housed individually in the same room (Razzoli et al., 2018). During the psychosocial stress phase, the intruder mice are exposed to the dominant resident mice for 10 min each day and, for the rest of the day, to sensory contact housing (enabled by a wire mesh partition bisecting the cage into two symmetrical compartments, each with food and water available ad libitum). In the aging phase, mice were housed in a sensory contact, thus experiencing a continued degree of threat stress related to the previous encounters (Razzoli et al., 2018). The results demonstrate that LCPS mice characterised by receiving high aggression, while exhibiting low aggression or identified as subordinate (equivalent to low social rank, a model of low socio‐economic status), showed lower survival probability and increased markers of cellular senescence compared to dominant mice. Remarkably, subordinate, but not dominant mice, developed spontaneous early‐stage atherosclerotic lesions in the aortic sinuses characterised by significant immune cell infiltration and sporadic rupture and calcification (Razzoli et al., 2018). These findings may offer a mechanistic explanation to the established association between low socio‐economic status and lower survival in humans.

### 3.4 Social isolation stress

Social isolation and loneliness are common sources of chronic psychosocial stress in humans and demographic changes have increasingly put more people at risk for loneliness in modern society (Xia & Li, 2018). Moreover, social isolation and loneliness represent a health risk factor comparable to cigarettes smoking and alcohol consumption. They increase mortality risk more than obesity and physical inactivity do. Molecular mechanisms underlying the increased disease risk induced by loneliness and social isolation are poorly understood (Xia & Li, 2018). Whether dysregulation of circadian rhythm is involved in the pathogenesis is unclear. There are first hints that social isolation in mice may influence the circadian clocks. For instance, post‐weaning social isolation (for 6 weeks starting at the age of 3 weeks), changed the pattern of CLOCK expression in gonadotropin‐inhibitory hormone neurons in the dorsomedial hypothalamus (Teo, Soga, & Parhar, 2017). Social isolation in adult mice led to decrease of mouse activities, phase advance of rhythm and down‐regulation of clock gene expression (Yu et al., 2018). The significance of these changes for cardiovascular health is yet unknown. Studies conducted under the context of cardiovascular pathology are still missing.

### 3.5 Pathways involved in stress‐induced circadian changes

Although the mechanisms involved in stress‐induced circadian changes are not known in detail, the involvement of the sympathetic nerve system and the hypothalamic–pituitary–adrenal (HPA) axis are highly likely (Figure 3; Tahara, Aoyama, & Shibata, 2017). Both social defeat stress (Tahara et al., 2015) and social isolation (Xia & Li, 2018) lead to the activation of the HPA and sympathetic–adrenal–medullary axes. Elevated circulating levels of corticosterone were observed after a single social defeat stress in male mice (Razzoli et al., 2014). Chronically maintained HPA hyperactivation was also evident in chronic social defeat as well as in lifelong social defeat models (Razzoli et al., 2014; Razzoli et al., 2018).

Glucocorticoids regulate the expression of several circadian genes via binding to the glucocorticoid response elements in the promoter regions (Tahara et al., 2017). Indeed, injection of dexamethasone at ZT4 for three consecutive days also induced phase entrainment of peripheral PER2 rhythms in the liver, kidney and submandibular gland (Tahara et al., 2015). These findings indicate that adrenal hormones represent key factors maintaining circadian rhythms in peripheral tissues *in vivo* (Tahara et al., 2017). Using non‐classical signalling, GR dimers are formed upon disruption of GR‐heat‐shock proteins, leading to activation of kinases such as PI3K, AKT or MAPK, independent of genomic events (Koch, Leinweber, et al., 2017).

Moreover, noradrenaline or adrenaline has been shown to induce circadian gene expression (Terazono et al., 2003). Treatment of mice with noradrenaline or adrenaline also caused phase advance of bioluminescence rhythms in peripheral tissues (Tahara et al., 2015), indicating a role of sympathetic activation in stress‐induced phase changes in peripheral clocks.

### 3.6 Noise

Aircraft noise caused endothelial dysfunction in healthy subjects (Schmidt et al., 2013) and increased BP in patients with established coronary artery disease (Munzel, Schmidt, et al., 2018). In laboratory mice, around‐the‐clock exposure to aircraft noise led to oxidant stress, vascular dysfunction, and elevation of BP (Kroller‐Schon et al., 2018; Munzel et al., 2017). These effects were absent or less pronounced in Nox2‐knockout mice, indicating a crucial role of NADPH oxidase‐mediated oxidative stress in the noise‐induced vascular phenotypes (Kroller‐Schon et al., 2018). Interestingly, dysregulation of the peripheral circadian clocks seems to be involved in the vascular pathology cause by noise. The effect of sleep phase noise exposure was stronger than awake phase noise exposure (Kroller‐Schon et al., 2018). Around‐the‐clock exposure to aircraft noise‐induced transcriptional changes in circadian genes in the aorta, heart and kidney.

### 3.7 Translation to humans

Disruption of circadian rhythm may promote the pathogenesis of a variety of disease including cardiovascular diseases (Morris, Purvis, Hu, & Scheer, 2016; Van Laake, Luscher, & Young, 2018). Circadian misalignment is a condition highly prevalent in shift workers. Higher blood glucose and insulin levels as well as elevated BP were observed in human subjects even only after a short circadian misalignment protocol (28 hr a day for 8 days; Scheer, Hilton, Mantzoros, & Shea, 2009). In another study, short‐term circadian misalignment (12‐hr inverted behavioural and environmental cycles for 3 days) per se increased BP and inflammatory markers (Morris et al., 2016). Consistent with these findings, shift workers show higher incidence of obesity, type II diabetes, hypertension, coronary heart disease and ischaemic stroke (Koch, Leinweber, et al., 2017).

## 4 ENVIRONMENTAL POLLUTION EFFECTS ON OXIDATIVE STRESS, CIRCADIAN CLOCK, AND CARDIAC HEALTH/DISEASE

### 4.1 Heavy metals effects on oxidative stress and cardiovascular health/disease

As outlined in a previous review article (Cosselman et al., 2015), there is substantial evidence from epidemiological and experimental data suggesting that heavy metals (e.g. cadmium, Tellez‐Plaza, Jones, Dominguez‐Lucas, Guallar, & Navas‐Acien, 2013, a systematic review of 31 studies and for lead, a systematic review of 12 studies, Navas‐Acien, Guallar, Silbergeld, & Rothenberg, 2007) and metalloids (e.g. arsenic, a systematic review of 12 studies, Moon, Guallar, & Navas‐Acien, 2012) can trigger cardiovascular diseases. Details on specific cardiovascular actions of different heavy metals can be found in the Supporting Information.

### 4.2 Environmental chemicals (e.g. pesticides) effects on oxidative stress and cardiovascular health/disease

Environmental chemicals with health effects comprise, among others, polychlorinated biphenyls (PCBs including dioxins), polybrominated diphenyl ethers (PBDEs), perfluorocarboxylic acids (PFCAs), perfluorooctanesulfonate (PFOS), benzene, and bisphenol A (BPA) that originate from industrial processes (e.g. material additives or combustion products) and pesticides (used to optimise crop yields by prevention of pest infestation). These compounds have proven adverse health effects mediated by induction of oxidative stress, inflammatory conditions or epigenetic regulations (e.g. via microRNAs; Miguel et al., 2018). Oxidative stress and adverse regulation of microRNAs (e.g. miR‐191/‐132/‐1537/‐21/‐31/‐126/‐221/‐222/‐188/‐34/‐362/‐338/‐155) result, besides cancer, in endothelial dysfunction, atherosclerosis, apoptosis, NASH, obesity and other cardiometabolic complications (Miguel et al., 2018), as well as neurodegenerative disorders (Drechsel & Patel, 2008). Details on specific cardiovascular actions of different environmental chemicals such as pesticides can be found in the Supporting Information.

### 4.3 Air pollution by particulate matter effects on oxidative stress and cardiovascular health/disease

The cardiovascular effects of air pollution are well characterised demonstrating vascular (endothelial) dysfunction, vascular inflammation and, in the longer run, the development of atherosclerosis (Rao et al., 2014). In addition, exposure to particulate matter increases oxidative stress within the vasculature and increases the sensitivity to vasoconstrictors (Ying et al., 2015). The impact of particulate matter and diesel exhaust on oxidative stress pathways and inflammation was reviewed in full detail in two recent articles within the forum issue “Oxidative stress and redox signaling induced by the environmental risk factors mental stress, noise and air pollution” (Munzel, Gori, et al., 2018). As outlined in a recent review article (Cosselman et al., 2015), around 7% of non‐fatal MIs and 18% of sudden cardiac deaths, are potentially triggered by exposure to road traffic‐dependent air pollution, representing comparable numbers to those published for the contribution of the major traditional and modifiable risk factors smoking, poor diet or obesity to cardiovascular morbidity and mortality. More details on specific cardiovascular actions of air pollution as well as beneficial effects of mitigation measures can be found in the Supporting Information.

### 4.4 Environmental pollution effects on circadian clock

The aforementioned link between environmental pollutants and oxidative stress already implies that these risk factors will affect the circadian clock, at least by redox regulatory mechanisms. A general review on the impact of environmental exposures to heavy metals and their impact on circadian clock, including a detailed discussion on potential mechanisms was recently published (Parmalee & Aschner, 2017). Alterations induced by chronic lead exposure on the cells of circadian pacemaker of developing rats were reported (Rojas‐Castaneda et al., 2011). Strong evidence exists for disruption of circadian rhythm by cadmium, which is termed “cadmium chronotoxicity” in the literature, could be partly prevented by antioxidant co‐therapy (suggesting a role for oxidative stress) and was reviewed extensively (Lafuente, 2013). In addition, copper overload may have adverse effects on circadian clock (Handy, 2003). More details can be found in the Supporting Information.

According to a study in flies, different pesticides showed consistently varying LD_50_ values at different daytimes, which correlated well with the diurnal expression profiles of xenobiotic metabolising genes, indicating that toxicity of pesticides is connected with the circadian rhythm in *Drosophila* flies (Beaver et al., 2010; Hooven, Sherman, Butcher, & Giebultowicz, 2009). Vice versa, it was shown that endocrine‐disrupting chemicals lead to dysregulation of the endogenous circadian clock, particularly in BPA‐exposed fish liver tissue, which may be explained by the presence of several xenobiotic response elements in the promoter regions of *Bmal1*, *Cry1*, *Cry2* and *Per2* (Rhee et al., 2014). The preclinical and clinical evidence for a crosstalk between environmental organic chemicals and circadian pathways as well as the underlying mechanisms (e.g. involvement of aryl hydrocarbon receptor‐dependent detoxification; Haarmann‐Stemmann & Abel, 2006) were reviewed in detail (Lim, Currie, Orphanides, & Moggs, 2006; Prokkola & Nikinmaa, 2018). More details can be found in the Supporting Information.

According to a recent review article, air pollution exposure causes changes in sleep/wake pattern and thereby increases the risk for vascular and cardiometabolic diseases; strikingly, several pulmonary and cardiovascular functions are affected that follow circadian rhythmicity (Haberzettl, 2018). Air pollution is also mentioned as a factor of chronobiologic aspects of venous thromboembolism (Fantoni, Dentali, & Ageno, 2017). Airborne particulate matter was also implicated in redox regulation of circadian molecular clock in chronic airway diseases (Sundar, Sellix, & Rahman, 2018). Similar considerations were made for exposure to ambient reactive gases such as ozone for which an intact circadian clock was proposed as a protective mechanism to pollution induced skin and keratinocytes damage (Benedusi, Frigato, Beltramello, Bertolucci, & Valacchi, 2018). Chronic ambient fine particulate matter (PM2.5) exposure was previously shown to trigger various adverse health outcomes, including atherosclerotic plaque progression (Sun et al., 2005), vascular oxidative stress and inflammation (Kampfrath et al., 2011). More recently, it was shown that PM2.5 exposure caused increased levels of stress hormone metabolites, 18‐oxocortisol and 5a‐tetrahydrocortisol and altered the levels of circadian rhythm biomarkers including melatonin, retinal and 5‐methoxytryptophol (Xu et al., 2019). In comparison to filtered air, ambient air particle exposure caused down‐regulation of key clock genes (*Per1*, *Per2*, *Per3*, *Rev‐erbα* and *Dbp*) and up‐regulation of *Bmal1*, in both pregnant rats and their offspring in the unfiltered group, which was associated with significant histological evidence of perivascular/alveolar inflammation and oxidative stress in the lungs (Song et al., 2017). In line with these data, a number of cardiovascular parameters that are known to be controlled by circadian clock are impaired by particulate matter exposure. Examples are impairment of nocturnal BP dipping and daytime urinary sodium excretion by short‐term increase in particulate matter levels (Tsai et al., 2012) or rapid changes in nocturnal heart rate variability (Lee et al., 2016). More details can be found in the Supporting Information.

## 5 MITOCHONDRIA AND ENVIRONMENTAL CHANGES

Mitochondria play a central role in cardiovascular disease development, especially in myocardial ischaemia/reperfusion injury (Hausenloy et al., 2017). However, cardioprotective interventions (conditioning strategies, either mechanical or pharmacological) also rely on intact mitochondrial function (Boengler, Lochnit, & Schulz, 2018). Importantly, many environmental changes impact on cardiac mitochondria especially under stress conditions, potentially rendering the cardiomyocytes less resistant towards ischaemia and reperfusion injury.

### 5.1 Circadian rhythmicity and mitochondrial function

Genes encoding for key proteins (citrate synthase, mitochondrial thioesterase 1 and uncoupling proteins) involved in mitochondrial metabolism and function showed a diurnal variation, a finding lost under pathophysiological conditions such as pressure overload or hypoxia (Young, Razeghi, Cedars, Guthrie, & Taegtmeyer, 2001). In isolated rat hearts, contractile performance, carbohydrate oxidation and oxygen consumption were greatest in the middle of the night, with little variation in fatty acid oxidation (Young et al., 2001). Hearts were isolated either in the middle of the light phase (ZT6) or the middle of the dark phase (ZT18). Similarly, peroxiredoxin III, the most abundant and efficient hydrogen peroxide‐eliminating enzyme in mitochondria and sulfiredoxin undergo antiphasic circadian oscillation in mitochondria (Rhee et al., 2014). Therefore, violation of diurnal cycle was accompanied by the intensification of lipid peroxidation process and marked with a reduced activity of antioxidant system enzymes and the activity of the enzymes involved in adenosine triphosphate synthesis in mitochondria was reduced (Kuchukashvili et al., 2011).

Interestingly, deletion of circadian rhythm in cardiomyocytes (specific circadian clock mutant mouse) increased myocardial oxygen consumption and fatty acid oxidation rates, whereas cardiac efficiency was decreased. These observations were associated with no alterations in mitochondrial content or structure and only a modest mitochondrial dysfunction (Bray et al., 2008). Similarly, there were no baseline differences in cardiac mitochondrial function between wild type mice and mice with deletion of the circadian rhythm gene mPer2 (mPer2‐M), but following ischaemia and reperfusion, wild type mice exhibited a marked decrease in maximal oxygen consumption supported by complex I‐mediated substrates, whereas mPer2‐M mice did not, despite no difference in complex I content. Moreover, cardiac mitochondria from wild type mice exhibited a very robust increase in ADP‐stimulated oxygen consumption in response to exogenously added cytochrome *c*, along with a high rate of ROS production, none of which was exhibited by cardiac mitochondria from mPer2‐M following ischaemia and reperfusion (Virag et al., 2013). In contrast, heart‐specific ablation of the circadian clock gene Bmal1 resulted in cardiac mitochondrial defects that include morphological changes and functional abnormalities, such as reduced enzymatic activities within the respiratory complex. Mice without cardiac Bmal1 function show a significant decrease in the expression of genes associated with the fatty acid oxidative pathway, the tricarboxylic acid cycle and the mitochondrial respiratory chain in the heart and develop severe progressive heart failure with age. Importantly, similar changes in gene expression related to mitochondrial oxidative metabolism were also observed in wild type mice subjected to chronic reversal of the light–dark cycle (Kohsaka et al., 2014).

### 5.2 Air pollution and mitochondrial function

The harmful effects of particulate matter with an aerodynamic diameter of <2.5 μm (PM2.5) and its association with acute coronary syndrome has gained increased attention in recent years. In rats, PM2.5 exposure induced pathological changes and ultra‐structural damage in hearts such as mitochondrial swelling and cristae disorder. Furthermore, PM2.5 exposure significantly increased specific mitochondrial fission/fusion gene (optic atrophy protein 1, mitofusin 1, dynamin‐related protein 1 and fission‐mediator protein 1) expression in rat hearts (Li et al., 2015). In mice, intranasally instilled residual oil fly ash (ROFA) reduced mitochondrial respiration predominantly through decreased complex II activity. In addition, mitochondria were depolarised and adenosine triphosphate production was reduced. Even though basal contractility was not modified in isolated mice hearts, they failed to properly respond to isoprenaline in ROFA‐exposed mice (Marchini et al., 2013). Interestingly, infliximab (a TNF‐α antibody) pretreatment attenuated impaired mitochondrial function in ROFA‐exposed mice (Marchini et al., 2015). Rats exposed to particulate matter exhibited no apparent inhibition of mitochondrial function during oxygenated conditions, but significantly greater myocardial mitochondrial injury was detected following ischaemia and reperfusion compared to untreated rats characterised by mitochondrial swelling and fusion (Golomb et al., 2012). Furthermore, in exposed male Sprague–Dawley rats, a significant increase in mitochondrial transition pore opening, leading to decreased mitochondrial function, was identified following exposure (Nichols et al., 2015). More examples can be found in the Supporting Information.

### 5.3 Noise exposure and mitochondrial function

Loud noise exposure (100 dBA) causes marked morphological alterations of the heart, including development of myocardial and perivascular fibrosis and a reduction of cardiac connexin 43 content in rats (Antunes et al., 2013). Additionally, a considerable number of enlarged mitochondria and some lipofuscin granules were observed (Antunes et al., 2013). In rat cardiomyocytes, the exposure to loud noise (for 12 hr) caused a significant increase of DNA damage accompanied by swelling of mitochondrial membranes, dilution of the matrix, and cristolysis. These alterations were accompanied by increased in situ noradrenaline levels and utilisation (Lenzi et al., 2003). Noradrenaline is the major substrate of monoamino oxidase A (MAO‐A) located at the outer mitochondrial membrane and ROS produced by MAO‐A led to the accumulation of p53 in the cytosol where it inhibited parkin, an important regulator of mitophagy, resulting in mitochondrial dysfunction (Manzella et al., 2018). Enlarged mitochondria were markedly positive for calcium accumulation and less calcium tolerant as opening of the mitochondrial permeability transition occurred more rapidly (Salvetti et al., 2000).

Taken together, many environmental changes impact on cardiac mitochondria especially under stress conditions, potentially rendering the cardiomyocytes less resistant towards ischaemia and reperfusion injury.

## 6 IMPACT OF DYSREGULATED CIRCADIAN CLOCK ON THE CARDIOVASCULAR SYSTEM

### 6.1 The circadian clock in cardiovascular cells

In the heart, approximately 13% of genes and 8% of proteins shows circadian rhythmic expression throughout the day (Thosar, Butler, & Shea, 2018). Oscillating circadian genes influence the activity of endothelial cells, fibroblasts and vascular smooth muscle cells (VSMCs; Takeda et al., 2007) and help to maintain normal cardiovascular physiology.

Endothelial cells become more susceptible to injury with polymorphisms in *Bmal1*, increasing the risk of developing hypertension (Durgan & Young, 2010). Fibroblasts display oscillations in circadian clock genes when grown in cell culture (Crnko et al., 2019). VSMCs have been shown to have synchronous, oscillating circadian rhythms throughout the day, stimulated by the induction of angiotensin II, which is a Zeitgeber to VSMCs (Nonaka et al., 2001). Blood coagulation markers display night and day patterns. Thrombomodulin, a gene that regulates intravascular coagulation in vascular endothelial cells, displays circadian oscillations. Oscillation of thrombomodulin gene expression was abolished in clock mutant mice and was altered by temporal feeding restriction, suggesting that its expression is under the control of the peripheral clock (Takeda et al., 2007). Our group found oscillating clock genes in human fetal and adult cardiac progenitor cells. Circadian rhythmicity influenced their ability to proliferate, tolerate different stressors, and secrete paracrine factors (Du Pre et al., 2017). Finally, normal vascular function in the heart is dependent on the circadian clock. Animal studies have shown that mice with mutations in *Per2* suffer from endothelial dysfunction, characterised by a decreased production of NO and vasodilatory PGs and increased production of vasoconstrictors (Viswambharan et al., 2007). Mice with mutant *Per2* display impaired angiogenesis, increased senescence marker expression on their endothelial progenitor cells (EPCs) and impaired blood flow when responding to ischaemia (Wang et al., 2008). More examples can be found in the Supporting Information.

Besides being regulated via neurohumoral input, heart rate is also under direct control of the cardiomyocyte circadian clock. Radio‐telemetry studies have shown that cardiomyocytes from clock mutant mice have bradycardia and reduced diurnal variations in heart rate compared to WT mice. Cardiac power was higher in WT mice compared to cardiomyocyte clock mutant mice, with no alterations found in mitochondrial function in the cardiomyocytes (Bray et al., 2008).

Functioning circadian rhythms also play a part in protecting the heart from stress‐induced damage. Noradrenaline functions as a Zeitgeber for cardiomyocytes, induces the circadian expression of dehydrogenase kinase isozyme or uncoupling protein‐3, which protects cardiomyocytes against ROS and stress‐induced myocardial damage (Wang et al., 2010). Arterial blood pressure (BP), essential in maintaining normal cardiovascular physiology, displays a rhythm, peaking mid‐morning and decreasing slowly throughout the day (Floras et al., 1978). A rise in BP increases the afterload on cardiomyocytes, stimulating the release of atrial natriuretic peptide (ANP), a cardioprotective agent that protects cardiomyocytes from damage caused by the increase in afterload (Takeda & Maemura, 2015). Nondipper is a term to describe people who do not exhibit a dip or decrease in the BP at night time. This cohort of people are more likely to have left ventricular cardiac hypertrophy and have an increased risk of dying from cardiovascular events (Fagard et al., 2009) compared to individuals who have normal BP or diurnal/dipping hypertension, further proving that having normal diurnal patterns in BP can contribute to normal cardiovascular physiology (Reitz & Martino, 2015).

### 6.2 Circadian rhythms in cardiovascular pathophysiology

**FIGURE 4:**
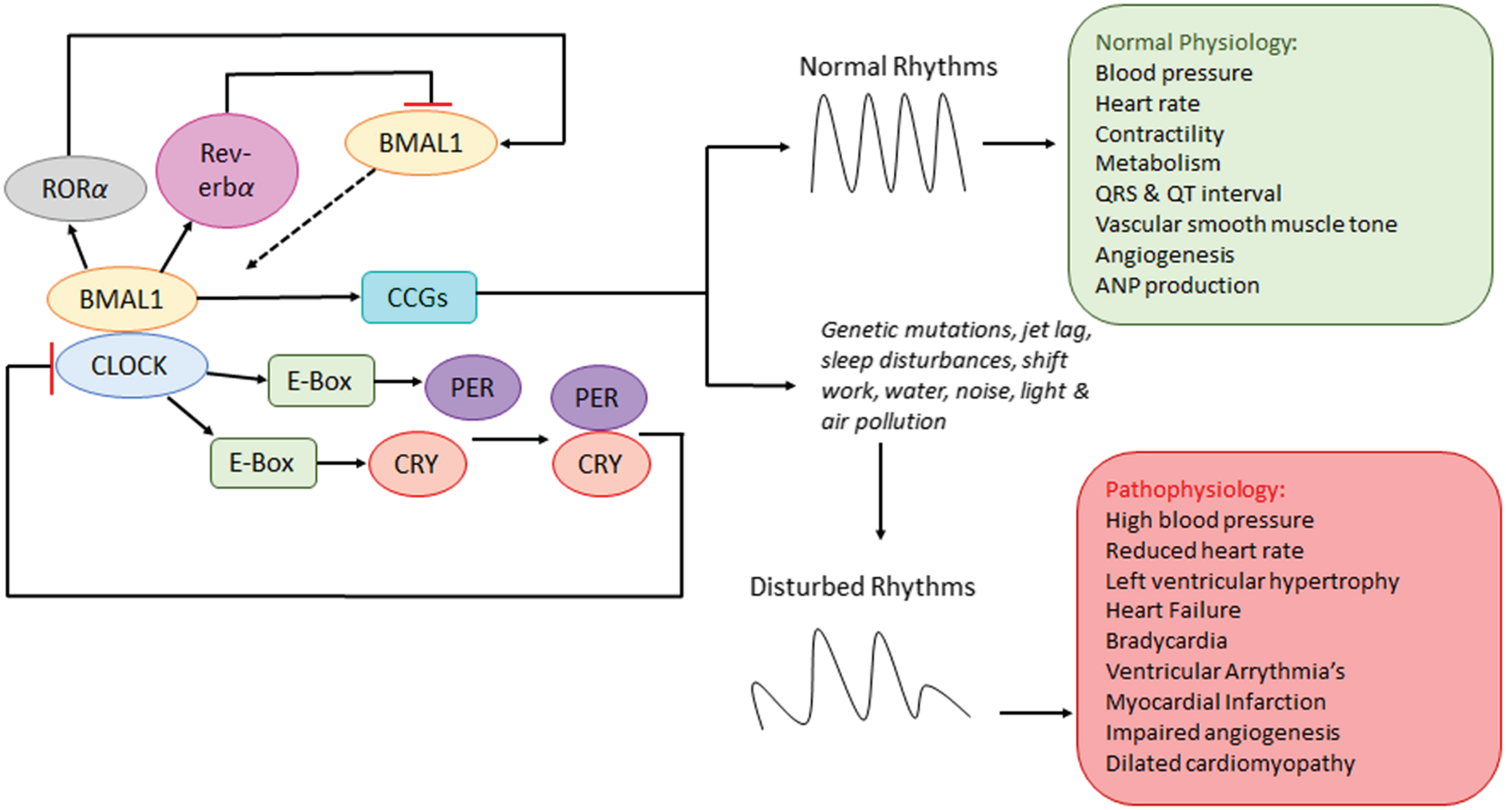
Impact of dysregulated circadian clock on the cardiovascular system. The circadian rhythm is tightly controlled by positive and feedback mechanisms within the tissues and cells of the heart to maintain normal cardiovascular physiology. In mammals, the transcription factors BMAL1/CLOCK activate the expression of PER1/2 and CRY1/2 clock proteins, which feedback repress BMAL1/CLOCK to close the 24‐hr cycle. Circadian oscillation of BMAL1 is regulated by the nuclear receptors RORα (activator) and Rev‐erbα (repressor; Merbitz‐Zahradnik & Wolf, 2015). Experimental and clinical evidence suggests a significant link between circadian rhythms and the development or progression of cardiovascular diseases (Rabinovich‐Nikitin et al., 2019). Genetic mutations in clock genes (PER, CRY, BMAL1, and CLOCK; Ko & Takahashi, 2006), jet lag, sleep disturbances, shift work (Reitz & Martino, 2015), water, noise, light and air pollution (Landrigan et al., 2018) are thought to play a role in disturbing circadian rhythms and increases the risk of developing cardiovascular pathology

Cardiovascular disease is one of the leading causes of morbidity and mortality in the world. There is ample experimental and clinical evidence suggesting a significant link between circadian rhythms and the development or progression of cardiovascular diseases (Rabinovich‐Nikitin, Lieberman, Martino, & Kirshenbaum, 2019; Figure 4). Synchronous circadian rhythms are essential for the normally functioning cardiovascular system (Crnko et al., 2019). However, in some pathological states, the circadian clock may be disrupted. There is growing epidemiological evidence to suggest that environmental and external factors such as depression, anxiety, social isolation, shift work, noise and air pollution may activate oxidative stress, increase autonomic nervous system imbalance and vascular dysfunction, which can lead to the development of cardiovascular diseases. The onset of different cardiovascular diseases, such as MI, ventricular arrhythmias and sudden cardiac death displays diurnal variations, in addition the outcome may also be related to the time of onset (Reitz & Martino, 2015).

Shift workers and people suffering from sleep apnoea are more likely to develop cardiovascular diseases due to the disturbances in their sleep patterns, which dysregulates their circadian rhythms by altering the heterodimerisation pattern of *Bmal1‐CLOCK* (Reitz & Martino, 2015). It has also been postulated that changes in the maximum and minimum input into the cardiac vagal autonomic and sympathetic nervous control contributes to the massive increase in cardiovascular diseases in shift workers (Furlan et al., 2000). Changes in mental and physical activity during the day influence the circadian rhythms in BP (Degaute, van de Borne, Linkowski, & Van Cauter, 1991). More examples can be found in the Supporting Information.

As mentioned previously, acute MI (AMI) is more likely to occur in the early hours in the morning (Takeda & Maemura, 2011). The underlying mechanisms of this observation involve both the central and peripheral circadian clocks. Heart rate, vasoconstriction of blood vessels and BP are all elevated in the morning, increasing the energy demand and decreasing the blood flow to the heart (Kurnik, 1995). Plasminogen activator inhibitor‐1 and other platelet surface markers are also elevated in the morning, which also contributes to the increased incidence of AMI in the morning (Tofler et al., 1987). Environmental factors have shown to increase the incidence of AMI. The shift from winter to summertime has shown to disrupt the circadian rhythms and influence the amount and quality of sleep a person gets. Incidence of AMI significantly increased 3 weeks after this external time reset, with a more pronounced difference in women than in men (Janszky & Ljung, 2008). Clock mutant mice exhibited altered immune cell infiltration post‐MI, and decreased blood vessel formation was observed 1 week post‐MI in the proliferative phase and worsened outcomes (Alibhai et al., 2014). More details can be found in the Supporting Information.

Desynchronised circadian rhythms have been shown to cause dilated cardiomyopathies. Ubiquitous knockout of *Bmal1* causes dilated cardiomyopathy in mice; a cardiomyocyte‐specific *Bmal* knockout model proved the role of the cardiomyocyte circadian clock in maintaining rhythmicity of the transcriptome (Young et al., 2014). Hamsters that have a mutant allele of the circadian clock related gene casein kinase 1 epsilon (CKIƐ; Hurd & Ralph, 1998) have reduced circadian periodicity from 24 to 22 h and increased incidence of cardiac pathology.

## 7 APPROACHES RELATED TO ENVIRONMENTAL RISK FACTORS AND “CHRONO” THERAPY FOR CARDIOPROTECTION

Whereas it is clear that interaction of classical risk factors, co‐morbidities and co‐medications play an important role for cardio‐protection (Ferdinandy, Hausenloy, Heusch, Baxter, & Schulz, 2014), we here want to complement this concept by therapeutic approaches targeting dysregulation of the circadian rhythm by environmental factors. It is clear that the circadian clock is crucial in maintaining health. Lifestyle plays a huge role in disrupting circadian rhythms and therefore predisposing individuals to developing cardiovascular diseases. Desynchronization of the circadian rhythm can occur if individuals have irregular sleeping patterns or have a jet setting lifestyle with frequent flying, constantly working in changing shift patterns and being exposed to different light–dark environments, which leads to an increased risk of developing neurological disorders and cardiovascular disease (Khan et al., 2018). *Per1* and *Per2* shift faster compared to *Clock* when an individual is phase shifting and causes disruption of the circadian clock (Khan et al., 2018). Mutations in genes such as *Bmal1*, *Cry1*, *Cry2*, *Rora*, and *Clock* can alter sleep patterns (Ko & Takahashi, 2006). Obesity has been linked with *Cloc*k (Jagannath, Taylor, Wakaf, Vasudevan, & Foster, 2017), *PER2* (Englund et al., 2009), and *Bmal1* (Williams & Schwartz, 2005) mutations seen in both human and mouse models. The risk of developing diabetes is increased in shift workers as disturbances in circadian rhythms can result in disturbed blood glucose control and as a result increase insulin resistance (Gonnissen et al., 2013). More examples can be found in the Supporting Information.

**FIGURE 5:**
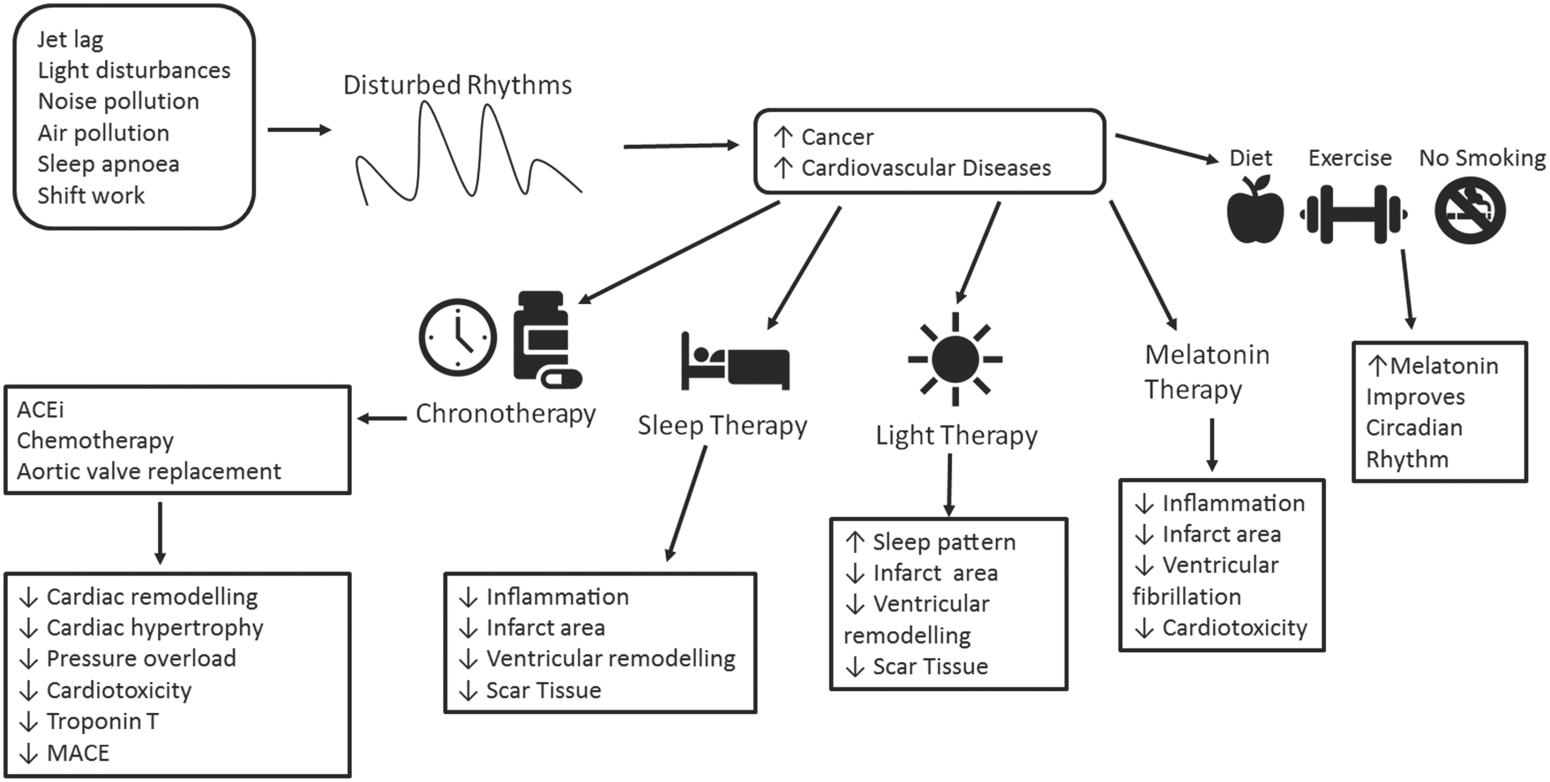
Approaches related to environmental risk factors and “chrono” therapy for cardioprotection. Environmental risk factors have shown to disrupt circadian rhythms and increase the risk of developing cancer and cardiovascular diseases. Chronotherapy is thought to reduce side effects and increase efficacy. Chronotherapy, sleep therapy, light therapy and melatonin administration can improve clinical outcomes after a cardiovascular event in both human and animal models

Using the circadian clock to our advantage when administering therapies may be beneficial for clinical outcomes (Figure 5). Chronotherapy is the study of rhythmic cycles such as circadian and seasonal cycles and administering drug therapies in optimised circadian delivery schedules to improve the efficacy and tolerance to drug therapies (Zhang, Levi, & Chang, 2018). Cancer is known to be a chronotherapeutic disorder that requires a high dose of chemotherapy to kill cancerous cells and in the process damage normal cells leading to toxic side effects that includes cardiotoxicity. Toxicity and efficacy of a drug may be dependent on what time of the day the drug is administered at. Chemotherapy drugs such as cisplatin, 5‐fluorouracil (5‐FU) efficacy and tolerability may therefore increase when administered in a time‐dependent manner (Ballesta, Innominato, Dallmann, Rand, & Levi, 2017). Cardiotoxicity is defined as the structural or functional damage done to the heart that can be caused by the exposure of the heart to chemotherapy and radiotherapy, the most serious consequence of this being the development of heart failure (Kang, 2001). Doxorubicin, an anthracycline known to cause cardiotoxicity, is known to cause apoptosis of cardiomyocytes *in vitro* in a time‐dependent manner (Du Pre et al., 2017). Besides being affected by the cellular (peripheral) circadian clock, cardiotoxicity may also be modified by circulating output of the central clock, such as melatonin (Zheng, Li, & Kros, 2018).

Performing operations at a certain time during the day can lead to improvements in patient outcomes. Major cardiac surgery, especially when on cardiopulmonary bypass, entails the risk of perioperative myocardial injury, left ventricular systolic impairment and heart failure (Montaigne et al., 2018). In a clinical study carried out to determine the time‐dependent variation in perioperative myocardial injury in patients undergoing aortic valve replacement, patients who received their operation in the morning had increased troponin T release and more adverse events compared to patients who had their surgery in the afternoon. The study also found that mice with *Rev‐Erbα* deletion or received antagonist treatment to the gene had reduced injury during their sleep to wake phase and increased expression of the gene CDKN1a/p21, involved in moderating ischaemia–reperfusion injury (Montaigne et al., 2018).

Maintaining a normal functioning circadian can improve outcomes in the clinical setting. Hospitals across Europe are now investigating the benefits of daylight therapy, by creating day and night skies in the hospital using LED light. A study carried out in Maastricht has shown that introducing this light therapy to patients resulted in improved sleep patterns in cardiac patients (Gimenez et al., 2017). In animal studies, altering the diurnal environment worsens their outcomes and increases inflammation in the infarct zone after a MI. In human patients post‐MI, maintaining a regular sleeping pattern to maintain functioning circadian rhythms may improve their outcomes (Sharma & Canty, 2014). Maintaining their circadian rhythms and giving them enough sleep can help with innate immune cell recruitment and limit ventricular remodelling and scar tissue formation (Yamashita et al., 2003).

It is important to note that young animals can adapt to changes in their external environment better than older animals (Chang & Guarente, 2013). Therefore, when setting up *in vivo* studies, one must consider the age of the animal being used. Preclinical animal studies are usually performed during the day with the lights are turned on. Therefore, experiments performed on mice should indicate that they were carried out during their inactive phase. To get a correct interpretation of the circadian clock, it is important to obtain data generated during the night in mice as this would be equivalent to humans during the day, which could potentially increase the success of translating preclinical data to the clinical setting (Lecour et al., 2014). Light settings could also be adjusted to coincide with the seasons in the outside environment (Jia et al., 2012). Therefore, when designing a study to assess the cardioprotective potential of a new therapy, it is important to take the time of the day into consideration to improve the efficacy and tolerability of the treatment. More examples can be found in the Supporting Information.

The common denominator with desynchronised circadian rhythms is that melatonin release is dysregulated. Melatonin is a pineal gland hormone that maintains regular circadian rhythms and is known to be a powerful anti‐hypertensive and antioxidant (Lochner, Huisamen, & Nduhirabandi, 2013). Melatonin is also a cardioprotective agent that reduces infarct size and decreases ventricular fibrillation after ischaemia–reperfusion, although clinical results have been conflicting (Lochner et al., 2013). Melatonin administered to AMI patients—independent of clock time—showed no improvement between left ventricular volumes, ejection fraction and infarct size compared to placebo (Dominguez‐Rodriguez et al., 2017). On the other hand, more time‐targeted therapy may have given different results and treating individuals with dysregulated circadian rhythms due to depression, jet lag and sleep disorders can re‐regulate circadian rhythms to help prevent the development of cardiovascular events (Srinivasan, Spence, Pandi‐Perumal, Trakht, & Cardinali, 2008).

## 8 CONCLUSIONS AND CLINICAL IMPLICATIONS

In conclusion, environmental factors such as noise, air pollution and mental stress are playing a role in dysregulating the circadian rhythm. Taking procedures such as implementing noise and air pollution regulations could reduce the risk of development of various cardiovascular diseases. Lifestyle changes to complement the circadian rhythm and therapies such as anti‐stress therapy, chronotherapy and melatonin treatment could reduce circadian rhythmic desynchronisation and prevent the development of cardiovascular diseases. As shown here, even normalising the cellular redox state under oxidative stress conditions or improving physiological functions of mitochondria could represent efficient therapies of dysregulated circadian rhythm. It is crucial to recognise the importance of circadian rhythms in maintaining normal physiology, both cardiovascular and neurological. More research involving tailoring therapies around the circadian rhythms will overcome the inconsistent results seen in clinical trials and can therefore make chronotherapy delivery to patients more practical for clinicians.

### 8.1 Nomenclature of targets and ligands

Key protein targets and ligands in this article are hyperlinked to corresponding entries in http://www.guidetopharmacology.org, the common portal for data from the IUPHAR/BPS Guide to PHARMACOLOGY (Harding et al., 2018), and are permanently archived in the Concise Guide to PHARMACOLOGY 2019/20 (Alexander et al., 2019).

## CONFLICT OF INTEREST

We certify that there is no conflict of interest with any financial organisations regarding the materials discussed in the manuscript.

## Supporting information

Data S1 Supporting Information
