# Supplementary material for: Influence of mental stress and environmental toxins on circadian clocks: Implications for redox regulation of the heart and cardioprotection
Source: Br J Pharmacol. 2020 Feb 4;177(23):5393–412. doi: 10.1111/bph.14949 (PMC7680009; doi:10.1111/bph.14949)
Supplement: Supplementary file 1 — Data S1 Supporting Information [file BPH-177-5393-s001.pdf]

Online supplemental information

Influence of mental stress and environmental toxins on circadian clocks - implications for redox regulation of the heart and cardioprotection

Huige Li[1]*, Aoife B. Kilgallen[2]*, Thomas Münzel[¶3,4], Eva Wolf[5,6], Sandrine Lecour[7], Rainer Schulz[8], Andreas Daiber[¶3,4]*, Linda W. Van Laake[2]*

[1] Department of Pharmacology, Medical Center of the Johannes Gutenberg University, Mainz, Germany; [2] Division Heart and Lungs and Regenerative Medicine Centre, University Medical Centre Utrecht and Utrecht University, Utrecht, Netherlands; [3] Center of Cardiology 1, Molecular Cardiology, Medical Center of the Johannes Gutenberg University, Mainz, Germany; [4] German Center for Cardiovascular Research (DZHK), Partner Site Rhine-Main, Mainz, Germany; [5] Structural Chronobiology, Institute of Molecular Physiology, Johannes Gutenberg University, Mainz, Germany; [6] Institute of Molecular Biology, Mainz, Germany; [7] Hatter Institute for Cardiovascular Research in Africa, University of Cape Town, South Africa; [8] Institute for Physiology, Justus-Liebig University Giessen, Giessen, Germany.

Running title: Exposome, circadian clocks and cardioprotection

[¶] **Address correspondence to:**

[¶] Prof. Dr. Andreas Daiber & Prof. Dr. Thomas Münzel, Universitätsmedizin der Johannes Gutenberg-Universität Mainz, Zentrum für Kardiologie 1 – Labor für Molekulare Kardiologie, Geb. 605 – Raum 3.262, Langenbeckstr. 1, 55131 Mainz, Germany, Phone +49 (0)6131 176280, Fax +49 (0)6131 176293, E-mail: daiber@uni-mainz.de or tmuenzel@uni-mainz.de

* These authors contributed equally and should be considered joint first and senior authors.

Extended "1. Introduction"

Extended information: 1.1 Environmental risk factors, disease burden and global mortality

The contribution of different risk factors to the global disease burden and their impact on life expectancy has shifted over the last 20 years from risks for communicable childhood diseases towards those for non-communicable adulthood diseases that are more frequently observed in the elderly (Lim et al., 2012; Murray et al., 2012). This shift is largely due to demographic changes, improved clinical prevention of childhood mortality, reductions in several preventable causes of death and a lower exposure to some risk factors. Socio-economic and scientific progress have led to improvements in water quality and sanitation, significant reductions in vitamin A and zinc deficiencies, and to lower exposures to particulate matter in households and the environment. There are large regional differences in the extent of these epidemiological and socio-economic changes and their impact on the importance of different risk factors, disease burden and mortality (e.g. poverty and childhood diseases continue to be the highest risk factors in sub-Saharan Africa) (reviewed in (Daiber et al., 2017)).

With the world population estimated to reach 9 billion by 2050, we face the daunting challenges of increasing environmental pollution with dramatic effects on public health. The Lancet Commission on pollution and health and the Global Burden of Disease (GBD) study identified air pollution (e.g. by particulate matter with a diameter of $\leq 2.5\mu m = PM_{2.5}$) as the leading health risk factor in the physical environment, followed by water pollution, soil pollution/heavy metals/chemicals and occupational exposures, however neglecting the non-chemical environmental health risk factors mental stress (Leka, Jain & Organization, 2010) and noise (Vienneau et al., 2015; Vienneau, Schindler, Perez, Probst-Hensch & Roosli, 2015) and light exposure as well as others (e.g. climate factors) (Collaborators, 2017; Landrigan et al., 2018). The Lancet Commission on pollution and health concluded that "Air pollution is the largest environmental cause of disease and premature death in the world today". The World Health Organization (WHO) even estimates that up to 12.6 million global deaths in 2012 were due to living in unhealthy environments (World Health Organization, 2016a; World Health Organization, 2016b). The leading role of environmental risk factors for public health becomes even more evident when it comes to

disease onset and progression, confirming the statement "Genetics load the gun, but environment pulls the trigger" by Francis Collins, director of the National Institute of Health (NIH). According to an estimation of the GBD study, disease caused by all forms of pollution were responsible for 268 million disability-adjusted life years (DALYs) - 254 million years of life lost and 14 million years lived with disability (DALYs & Collaborators, 2016). Despite the fact that 70% of pollution-related diseases are non-communicable diseases interventions against pollution are barely mentioned in the Global Action Plan for the Prevention and Control of Non-Communicable Diseases (Landrigan et al., 2018).

Robust epidemiological evidence for detrimental health effects of environmental risk factors and mechanistic insights into the underlying pathophysiology are mostly based on association studies (with only few controlled interventional studies) and exist for the environmental risk factors mental stress (e.g. social isolation, work stress) (Fransson et al., 2015; Nyberg et al., 2014; Rosengren et al., 2004), air pollution (e.g. particulate matter) (Beelen et al., 2014; Lelieveld, Haines & Pozzer, 2018; Munzel et al., 2018; Newby et al., 2015), noise exposure (e.g. traffic/occupational sources) (Kempen, Casas, Pershagen & Foraster, 2018; Munzel, Schmidt, Steven, Herzog, Daiber & Sorensen, 2018) and chemical pollution (e.g. heavy metals and pesticides) (Cosselman, Navas-Acien & Kaufman, 2015; GBD 2016 Risk Factors Collaborators, 2017; Navas-Acien, Guallar, Silbergeld & Rothenberg, 2007; Tellez-Plaza, Jones, Dominguez-Lucas, Guallar & Navas-Acien, 2013). Correlations are usually made between environmental exposures and health outcomes or biomarkers measured by different OMICs techniques (Ghezzi et al., 2018).

Extended "2. Oxidative stress, redox regulation and circadian clock"

Extended information: 2.2 Redox-regulatory mechanism in circadian clock

The structure of dCRY, a FAD-dependent circadian blue-light photoreceptor, revealed three cysteine residues (Cys337, Cys416, Cys523), which play a key role in the dCRY photoreaction and in phototransduction from the flavin adenine dinucleotide (FAD) cofactor to the regulatory protein tail. This light-induced electron transfer involves a chain of tryptophanes, cysteine residues and potentially nearby

located methionine residues that are all prone to oxidative modifications under oxidative stress conditions (and if reversible provide a basis for redox-regulation of this photoreceptor).

AMPK-dependent phosphorylation of mCRY1 leads to its proteasomal degradation by enhancing complex formation with FBXL3 (Lamia et al., 2009). Furthermore, AMPK causes degradation of period proteins by activation of casein kinase I (Jordan & Lamia, 2013; Lee & Kim, 2013). The mCRY1 crystal structure also visualized a MAPK phosphorylation site at Ser247. MAPK-dependent phosphorylation of mammalian CRY proteins (Ser247 of mouse mCRY1, Ser265 of mCRY2) negatively regulates transcriptional repression of circadian clock mediated gene expression (Sanada, Harada, Sakai, Todo & Fukada, 2004) and might also represent another stress response regulatory pathway of the circadian clock since MAP-kinases are activated under cellular stress conditions, are redox-regulated and initiate survival pathways but also apoptosis. Notably, the mutation of Ser247 in mCRY1 attenuated mCRY1 transcriptional repression activity in the circadian clock (Sanada, Harada, Sakai, Todo & Fukada, 2004), but also reduced FBXL3 binding in U2OS cells (Czarna et al., 2013). In addition, Cys363 and Cys412 residues of mCRY1 form an intramolecular disulphide bridge that causes a conformational change in a PER2-binding loop of mCRY1, but also may have important implications for mCRY1 interactions with the glucocorticoid receptor as well as glycaemic control (Czarna et al., 2013). In the crystal structure of the mCRY2 homologue, no disulphide bridge between Cys381 and Cys430 (corresponding to Cys363 and Cys412 of mCRY1) was observed (Xing et al., 2013). Instead, Cys430 of CRY2 forms an intermolecular disulphide bridge with Cys340 of FBXL3 in the mCRY2/FBXL3 complex structure, providing another example for high cysteine redox-reactivity in a mammalian clock protein (Xing et al., 2013) (**Figure 2**, middle left**, *main manuscript***).

Other redox-regulatory mechanisms in the circadian clock are based on NAD^+- and AMPK-dependent SIRT1 activation, where AMPK enhances SIRT1 activity by increasing intracellular NAD+ levels (Lee & Kim, 2013). In the presence of high NAD^+ levels, SIRT1 affects BMAL1/CLOCK dependent circadian gene regulation by deacetylating BMAL1-K537, histone H3 K9/K14 (Nakahata et al., 2008) and PER2 (Asher et al., 2008). Moreover, SIRT1 deacetylates PGC1α and thereby affects the

circadian clock via the PGC1α-RORs-RORE-axis, that causes activation of the CLOCK/BMAL1 complex (Lee & Kim, 2013). In addition, poly(ADP-ribose) polymerase-1 (PARP1) causes poly ADP-ribosylation of CLOCK in a NAD^+-dependent reaction, which inhibits DNA binding by CLOCK-BMAL1 and PARP1 activity is also connected to oxidative stress (Asher, Reinke, Altmeyer, Gutierrez-Arcelus, Hottiger & Schibler, 2010; Jordan & Lamia, 2013). Furthermore, the antioxidant forkhead box O (FOXO) transcription factors, which are insulin- / redox-regulated and control antioxidant gene expression, affect the circadian clock by modulating the sensitivity of the central clock to oxidative stress. This was shown by *foxo* mutants that exerted a rapid decline in rest/activity rhythms with age, thereby providing a link between insulin signalling, oxidative stress, aging, and circadian rhythms (Zheng, Yang, Yue, Alvarez & Sehgal, 2007). Moreover, circadian clock oscillation was irregular and the period was variable upon deletion of *foxo3* (Chaves et al., 2014). A BMAL1 binding site for FOXO3 was reported and FOXO3 down-regulation was associated with dysregulation of circadian clock gene expression in a murine model of aircraft noise exposure, whereas pharmacological activation of FOXO3 normalized endothelial function and vascular oxidative stress (Kroller-Schon et al., 2018). Other redox-regulated enzymes such as hypoxia-inducible factor 1α (HIF-1α) or heme oxygenase-1 (via carbon monoxide (CO)) also contribute to the redox-regulation of the circadian clock (Reinke & Asher, 2019) (**Figure 2, *main manuscript***). Heme oxygenase–derived CO attenuates DNA binding of the CLOCK(NPAS2):BMAL1 heterodimers, as does the excess of oxidized NAD(P) over reduced NAD(P)H (Rutter, Reick, Wu & McKnight, 2001).

Extended information: 2.3 Association of circadian clock with cellular redox state in health and disease

There is also strong evidence for redox regulation of circadian clock genes in chronic airway diseases (Sundar, Sellix & Rahman, 2018). More data on disease-triggered dysregulation of the circadian clock were generated by animal research in models of cardiac hypertrophy (Young, Razeghi & Taegtmeyer, 2001), diabetes (Young, Wilson, Razeghi, Guthrie & Taegtmeyer, 2002) and hypertension

(Naito et al., 2003) – all of these diseases are associated with endothelial dysfunction and increased vascular oxidative stress (Daiber et al., 2017). Likewise, there is obviously a general link between the circadian variation in blood pressure, vascular contraction and vascular redox state (Rodrigo & Herbert, 2018). Disturbance of diurnal rhythm caused changes in gene expression and early signs of cardiac hypertrophy (e.g. increased left ventricular end-systolic/diastolic dimensions) with rescue by diurnal resynchronization (Martino et al., 2007).

Extended "3. Mental stress effects on oxidative stress, circadian clock and cardiovascular health/disease"

Extended information: 3.1 Mental stress effects on oxidative stress and cardiovascular health/disease

Our group in Mainz can use the data of the Gutenberg Health Study (GHS), a population-based, prospective, single-center cohort study that aims at improving the individual risk prediction for diseases by inclusion of 15,000 individuals of the Rhine-Main area, with at least 2 follow-up visits and assessment of lifestyle, psychosocial factors, environment, laboratory parameters as well as the extent of the subclinical disease (Wild et al., 2012). Using the unique Gutenberg Health Study database, we were able to reveal that noise annoyance is associated with depression and anxiety in the general population – thereby providing a link between noise exposure, mental stress and cardiovascular risk (Beutel et al., 2016), also supported by association of mental stress/annoyance with atrial fibrillation (Hahad et al., 2018). A case of a Takotsubo cardiomyopathy, also known as the "broken heart disease" (Sinning, Keller, Abegunewardene, Kreitner, Munzel & Blankenberg, 2010), as a consequence of nighttime aircraft noise exposure was reported by our group (Munzel et al., 2016). Other studies have shown a direct link between mental stress and development of hypertension (Marvar et al., 2012). According to a recent WHO report, noise can contribute to cognitive impairment and adversely affect mental health (Clark & Paunovic, 2018a; Clark & Paunovic, 2018b). There is evidence that mental stress mediated cardiovascular and cognitive impairment is associated with increased oxidative stress (Meyer & Wirtz, 2018; Xia & Li, 2018).

Extended information: 3.3 Social defeat stress and circadian clocks

In a mouse model of repeated social defeat, the experimental male mice were exposed daily for 2h to social defeat over a time period of 19 days, either at the beginning of the light phase (ZT1-3) or at the beginning of the dark phase (ZT13-15). Single-housed mice served as control (Bartlang et al., 2012). Interestingly, the effects of this chronic/intermittent psychosocial stress were strongly dependent on the time of day of stressor exposure. Social defeat stress applied at light/inactive phase induced more beneficial adaptations (prolonged increase in adrenal weight and an attenuated adrenal responsiveness to adrenocorticotropic hormone (ACTH). In contrast, dark/active phase social defeat led to maladaptive signs such as reduced dark phase home-cage activity, flattening of the diurnal corticosterone rhythm, lack of social preference, and more pro-inflammatory status (Bartlang et al., 2012). Moreover, the SCN molecular rhythmicity was affected by repeated social defeat only when the stress was applied at dark phase, while light phase stress showed no effect on SCN rhythmicity. In contrast, the adrenal peripheral clock was mainly influenced by light phase social defeat (Bartlang, Oster & Helfrich-Forster, 2015; Bartlang, Savelyev, Johansson, Reber, Helfrich-Forster & Lundkvist, 2014).

The response of the adrenal clock to chronic social defeat stress did not habituate; the corticosterone level remained elevated after 14 days of stress (Razzoli, Karsten, Yoder, Bartolomucci & Engeland, 2014). The adrenal clock phase shift was associated with increased feed conversion efficiency, suggesting that the metabolic phenotype in the stressed mice may be (partly) caused by altered adrenal clock rhythmicity (Razzoli, Karsten, Yoder, Bartolomucci & Engeland, 2014). The locomotor activity rhythm and body temperature rhythm of stressed mice were not affected, indicating that the SCN clock was not changed by chronic social defeat stress (Razzoli, Karsten, Yoder, Bartolomucci & Engeland, 2014). Of note, the studies with social defeat stress are often performed only with male animals, because females have poorly developed territorial behaviour (Koch, Leinweber, Drengberg, Blaum & Oster, 2017). It is also important to note that there are general gender-specific differences in the GC system. Compared with male mice, female mice have both higher baseline and stress-induced plasma

corticosterone levels, and heavier adrenal glands (Koch, Leinweber, Drengberg, Blaum & Oster, 2017; Malisch, Saltzman, Gomes, Rezende, Jeske & Garland, 2007). Adrenalectomy led to disruption of PER2 oscillations in the bed nucleus of the stria terminalis (BNST) (Segall, Perrin, Walker, Stewart & Amir, 2006).

Extended "4. Environmental pollution effects on oxidative stress, circadian clock and cardiac health/disease"

Extended information: 4.1 Heavy metals effects on oxidative stress and cardiovascular health/disease

Increased blood levels of cadmium were associated with higher numbers of cardiovascular disease and mortality in a population-based cohort study in 4,819 Swedish adults (Barregard et al., 2016). An impact of blood lead levels on all-cause and cardiovascular mortality was found at substantially lower blood lead levels than expected by previously reported data (Menke, Muntner, Batuman, Silbergeld & Guallar, 2006). Increased cardiovascular disease, coronary heart disease, and stroke mortality upon chronic exposure to arsenic were also confirmed by a prospective cohort study in 3,575 American Indian men and women (Moon et al., 2013). According to the WHO and the Agency for Toxic Substances and Disease Registry (ATSDR), cadmium, lead and arsenic are also included in the top 10 environmental compounds of concern ((ATSDR), 2015). Similar to particulate matter, heavy metals and metalloids most probably interfere with essential physiological pathways that are associated with cardiovascular health or damage. A modulation of blood pressure, lipid metabolism, atherogenesis, and endothelial function was observed in response to these compounds (Prozialeck, Edwards, Nebert, Woods, Barchowsky & Atchison, 2008).

In the plasma, serum, and atherosclerotic lesions of mice treated with arsenic, increased levels of pro-inflammatory chemokines, cytokines, and markers of oxidative stress were observed (Srivastava, Vladykovskaya, Haberzettl, Sithu, D'Souza & States, 2009). Arsenic induces inflammatory signaling pathways, oxidative stress, inhibition of NO bioavailability, all of which promote proliferation of endothelial cells and smooth muscle cells, adhesion of immune cells and platelet aggregation and thereby

trigger cardiovascular disease (Barchowsky, Dudek, Treadwell & Wetterhahn, 1996; Kumagai & Pi, 2004; States, Srivastava, Chen & Barchowsky, 2009). Likewise, cadmium leads to vascular damage, endothelial dysfunction and atherosclerosis by oxidative mechanisms (e.g. by replacement of iron and copper in sulfur-complexes, promoting Fenton reactions) (Messner & Bernhard, 2010; Messner et al., 2009), interference with antioxidant responses (e.g. by disruption of zinc-sulfur complexes) (Cuypers et al., 2010) and inhibition of $^{\bullet}$NO-mediated vasodilation (Almenara et al., 2013). In addition, there is also evidence for adverse effects of heavy metals and metalloids on epigenetic regulation of gene expression (Cosselman, Navas-Acien & Kaufman, 2015). Similar reports indicate that lead contributes to oxidative stress, inflammation, endothelial dysfunction, and proliferation of vascular cells with adverse effects on heart-rate variability (Navas-Acien, Guallar, Silbergeld & Rothenberg, 2007; Vaziri, 2008). Overall, lead mimics many biological effects of cadmium (Cosselman, Navas-Acien & Kaufman, 2015). Of note, lead inhibits the $^{\bullet}$NO / soluble guanylate cyclase signaling pathway, stimulates the renin–angiotensin-aldosterone system and the sympathetic nervous system, and activates protein kinase C activity, all of which resemble strikingly the adverse effects of noise exposure on the cardiovascular system (Vaziri, 2008). The impact of environmental chemicals such as heavy metals on oxidative stress pathways and epigenetic gene regulation was reviewed in full detail in a recent article within the Forum issue "Oxidative stress and redox signaling induced by the environmental risk factors mental stress, noise and air pollution" (Miguel et al., 2018).

There are also limited data available on the adverse effects of other toxic metals (e.g. mercury, tungsten and antimony) on cardiovascular risk and disease (Navas-Acien, Silbergeld, Sharrett, Calderon-Aranda, Selvin & Guallar, 2005; Tyrrell, Galloway, Abo-Zaid, Melzer, Depledge & Osborne, 2013). However, a systematic review of epidemiological studies on the association between cardiovascular disease in adults and the environmental metals antimony, barium, chromium, nickel, tungsten, uranium, and vanadium summarized that, due to the limited number of reliable clinical studies, no final conclusion can be made (Nigra, Ruiz-Hernandez, Redon, Navas-Acien & Tellez-Plaza, 2016). A detrimental role for the development of cardiovascular disease is discussed for essential metals (e.g. iron, cobalt, copper, zinc

and selenium) since exposure to them is often far above those reported for the “toxic” metals. However, these toxic effects by the essential metals are not easily studied since their absorption and excretion is tightly regulated by transporters and storage mechanisms, and they usually show no serious accumulation.

Extended information: 4.2 Environmental chemicals (e.g. pesticides) effects on oxidative stress and cardiovascular health/disease

The mechanisms how the above mentioned environmental toxins induce oxidative stress, show a large variety but are mainly based on P450 chemistry, redox cycling, suppression of antioxidant enzymes and uncoupling of mitochondrial respiration, all of which has been extensively revised in the past (Drechsel & Patel, 2008; Stohs, 1990; Videla, Barros & Junqueira, 1990). Besides the well-documented impact on neurodegenerative disease progression (Cao, Souders Ii, Perez-Rodriguez & Martyniuk, 2018; Masuo & Ishido, 2011; Sanchez-Santed, Colomina & Herrero Hernandez, 2016), chronic exposure to these environmental toxins will ultimately contribute to the development of diabetes (Chevalier & Fenichel, 2015; Lind & Lind, 2018), hypertension (Han & Hong, 2016; Kahn & Trasande, 2018), arteriosclerosis (Lind & Lind, 2012; Ross, Matthews & Mangum, 2014) and cardiovascular disease incidence/mortality (Humblet, Birnbaum, Rimm, Mittleman & Hauser, 2008), as epidemiological studies show positive associations for these organic pollutants (POPs, e.g. dioxins or pesticides), plastic associated chemicals (PACs, e.g. BPA) with said diseases (Argacha, Mizukami, Bourdrel & Bind, 2019).

Extended information: 4.3 Air pollution by particulate matter effects on oxidative stress and cardiovascular health/disease

Despite the relatively low contribution of air pollution to the individual risk of a single human being, the cumulative cardiovascular risk conferred by chronic exposure of a large part of the population to ubiquitous air pollution (the cumulative global disease burden) even ranks above physical exertion, coffee and alcohol in a comparative risk assessment of the major triggers of myocardial infarction (Nawrot, Perez, Kunzli, Munters & Nemery, 2011). Only diet, high blood pressure, and smoking

represent more important cardiovascular risk factors for life years with severe illness and disability than air pollution (Yang et al., 2013). This is further supported by the fact that around 7 % of non-fatal myocardial infarctions (Nawrot, Perez, Kunzli, Munters & Nemery, 2011) and 18 % of sudden cardiac deaths (Hart, Chiuve, Laden & Albert, 2014), are potentially triggered by exposure to road traffic-dependent air pollution, representing comparable numbers to those published for the contribution of the major traditional and modifiable risk factors smoking, poor diet, or obesity to cardiovascular morbidity and mortality (Hart, Chiuve, Laden & Albert, 2014). Air pollution in the form of particulate matter is a well-established cardiovascular risk factor, also acknowledged by leading cardiac societies such as the American Heart Association (Brook et al., 2010). Air pollution impact on induction of cardiovascular oxidative stress is well accepted (Rao, Zhong, Brook & Rajagopalan, 2018; Wilson, Miller & Newby, 2018). Strikingly, the short-term pollution-control activities (mainly reduction in traffic and industrial air pollution) applied during the 2008 Beijing Olympic Games led to a 13–60 % reduction in the concentrations of air pollutants, which were perfectly mimicked by similar effects on biomarkers of inflammation, oxidative stress, and thrombosis in healthy adults (Huang et al., 2012; Kipen et al., 2010; Rich et al., 2012; Roy et al., 2014). Unfortunately, the beneficial effects quickly returned back to normal when the restrictions for air pollution were invalidated after the Olympic Games. Even more striking results were obtained when Tokyo decreased diesel emissions by new restrictive laws leading to a 44 % decrease in PM from traffic between 2003 and 2012. Osaka introduced similar laws in 2009. When comparing the mortality rates in Osaka with those in Tokyo, a striking decrease in cardiovascular mortality by 11 % among Tokyo's population was observed that was mainly due to a 10 % decrease in ischaemic heart disease mortality (Yorifuji, Kashima & Doi, 2016).

Extended information: 4.4 Environmental pollution effects on circadian clock

It was shown in mice that arsenic treatment for 6 months resulted in alterations of circadian rhythm, longer freezing time and escape latencies compared to the control group, all of which was associated with decreased ATP levels, impaired complex I activity and increased oxidative stress markers

in the hippocampus as well as more pronounced immune reactions in response to amyloid isoforms (Nino et al., 2019). Data mining and interaction network analysis of sources related to human bladder cancer revealed a correlation between environmental arsenic exposure and changes in circadian genes (Polo et al., 2017). Vice versa, in birds the plasma concentrations of the metals zinc, copper and molybdenum revealed diurnal fluctuations among a set of 14 elements indicating that uptake, storage and excretion of these elements may be also regulated by circadian clock (Rosenthal, Johnston, Shofer & Poppenga, 2005), which was also confirmed by levels of seven heavy metals (Pb, Zn, Cu, Cr, Cd, Hg, Mn) in plasma, erythrocytes and urine of metal workers (Yokoyama, Araki, Sato & Aono, 2000).

Similar evidence exists for polychlorinated biphenyls (PCBs) and their hydroxylated metabolites in Wistar rats, which have been shown to disrupt the circadian rhythm and cause dysregulation of down-stream peroxisome proliferator-activated receptor-signalling associated with adverse fatty acid metabolism (Ochiai et al., 2018). Treatment with wastewater, containing a sub-lethal mixture of pharmaceutical and personal care products (PPCPs), affected the circadian oscillation in the activity of male mosquitofish (Melvin, Buck & Fabbro, 2016). Interaction of xenobiotics and circadian clock was also reviewed in detail (Claudel, Cretenet, Saumet & Gachon, 2007).

Bisulphite sequencing of 407 newborns revealed epigenetic regulation of circadian genes in dependence of the regional PM2.5 exposure levels of their mothers during gestation (Nawrot et al., 2018). Epigenetic regulation was observed by altered placental methylation of CpG sites within the promoter regions of circadian genes *Npas2, Cry1, Per2* and *Per3* suggesting that PM2.5 exposure might affect placental processes and foetal development. Similar observations were made for methylation of the Clock gene in blood cells of free-living birds in dependence of PM10 exposure (Romano et al., 2017).

Extended "5. Mitochondria and environmental changes"

Extended information: 5.2 Air pollution and mitochondrial function

Smaller particles (nanoparticles) have also documented impact on mitochondria. Combustion- and friction-derived magnetic air pollution nanoparticles were present inside mitochondria in ventricular

cardiomyocytes, in endoplasmic reticulum (ER), at mitochondria-ER contact sites (MERCs) and intercalated disks. Left ventricular prion protein up-regulation occurred in these hearts (Calderon-Garciduenas et al., 2019) , which has been viewed to contribute to the cardiac mechanisms, antagonizing oxidative insults (Zanetti et al., 2014). Similarly, marked abnormalities in mitochondrial morphology were seen in dogs exposed to high concentrations of air pollutants including ultrafine PM. Unlike control samples where the mitochondrial cristae were closely packed, uniform and linear, exposed dogs had fragmented or missing cristae, with intra-mitochondrial lucent areas and an increase in fusion of multiple mitochondria producing giant mitochondria. Numerous nano-sized particles were observed attached to the abnormal cristae or in the electron lucent matrix (Villarreal-Calderon et al., 2013). In in vitro studies, 24-hour exposure to nano particulate matter increased the oxidation of mitochondrial DNA and decreased mitochondrial oxygen consumption rate (Breton et al., 2019). Diesel exhaust exposure induced an alteration in mitochondrial oxidative capacity with specific alteration in complex I of the respiratory chain after repeated exposures (Karoui et al., 2019).

Extended "6. Impact of dysregulated circadian clock on the cardiovascular system"

Extended information: 6.1 The circadian clock in cardiovascular cells

Maintaining proper metabolism in the heart is essential in maintaining its contractility to meet the daily demands and increased workload experienced during the active phase of the day. Fatty acids and glucose are the 2 main nutrients that maintain myocardial contractility (Taegtmeyer, 2000). Studies carried out in cardiomyocyte clock mutant (CCM) mice revealed that a huge number of genes involved in lipid (triglyceride) and glycogen metabolism are controlled by the circadian clock (Bray et al., 2008). Cardiomyocytes generate ATP during their active period to increase their contractility and store nutrients towards the end of their active phase for growth/repair during their rest phase (Thosar, Butler & Shea, 2018). This mirrors the triglyceride synthesis, where triglycerides levels display diurnal variation and peak in in the morning in WT mouse hearts and are completely absent in cardiomyocytes of CCM mice (Tsai et al., 2010). Glycogenolysis, driven by adrenaline occurs in a time-dependent manner in WT mice

but this oscillation is depressed in CCM mice (Bray et al., 2008) . CCM mice exhibit a reduced heart rate after exercise, compared to wild type (WT) mice that undergo the same intensity of exercise during their active phase (Bray et al., 2008). The propagation and generation of electrical signals in the heart has shown to be influenced by the circadian clock. There are diurnal variations of ions, notably Ca^{2+} and K^{+}, that are involved in the electrophysiology and excitation-contraction of the heart (Collins & Rodrigo, 2010; Jeyaraj et al., 2012). Gap junction proteins, such as connexin 40 involved in atrial-ventricular conduction, exhibit oscillatory expression in WT mice which is absent in the hearts of CCM mice (Bray et al., 2008).

Extended information: 6.2 Circadian rhythms in cardiovascular pathophysiology

There is growing epidemiological evidence to suggest that environmental and external factors such as depression can lead to the development of cardiovascular diseases (Hickie, Naismith, Robillard, Scott & Hermens, 2013) and preclinical data indicate that disturbed circadian rhythm represents an important determinant in heart disease (Martino et al., 2007). Shift workers will have resynchronised blood pressure rhythms 24 hours after a shift rotation (Chau et al., 1989). Patients that have sleep apnoea are found most commonly in airline crew and shift workers are more likely to develop cardiovascular diseases and show increased incidence of adverse cardiovascular events (Bradley & Floras, 2003; Furlan, Barbic, Piazza, Tinelli, Seghizzi & Malliani, 2000). Changes in the circadian autonomic system activity have shown to increase the risk of stroke, heart attack, ischemic heart disease and sudden death in these cohort of people (Furlan, Barbic, Piazza, Tinelli, Seghizzi & Malliani, 2000). Repeatedly altering the light/dark cycles of hamsters with cardiomyopathies significantly reduced their survival. Reducing the light/dark cycle from 24h to 20h of mice had worsened outcomes when they were exposed to a pressure overload cardiac hypertrophy model. Mice with disturbed rhythms had abnormal end-systolic and diastolic dimensions, reduced contractility and worsened left ventricular remodelling post-MI, with altered *Per2*, *Bmal1*, ANP, brain natriuretic peptide (BNP), and ACE expression (Martino et al., 2007).

Restoration of the altered circadian rhythms reversed the abnormal pathophysiology (Martino et al., 2007).

Electrophysiology parameters, such as the QT interval and the AV node, show diurnal variations throughout the day. The SCN drives rhythmic autonomic nerve activity and the expression of voltage gated K+ ion channels such as *Kv4.2* and *Kv1.5*, leading to oscillations in the refractory period of cells (Yamashita et al., 2003). *Bmal1* also regulates the expression of sodium ion channel *Scn5a*. Mice that have *Bmal1* deleted from their cardiomyocytes have a slower heart rate and prolonged QRS interval with increased arrhythmias (Steinbach, Glogar, Weber, Joskowicz & Kaindl, 1982). These oscillations ad to the observed oscillating incidence of cardiac arrhythmias such as ventricular tachycardia/ fibrillation (VT/VF), sudden death and atrial fibrillation, in addition to an increase in sympathetic nervous stimulation (Englund, Behrens, Wegscheider & Rowland, 1999; Willich, Maclure, Mittleman, Arntz & Muller, 1993). The QT interval has been described to be regulated by clock genes. The transcription factor kruppel-like factor 15 (Klf15), involved in activating the potassium channel, Kv channel-interacting protein 2 (KChIP2), which regulates the outward flow of K+ ions out of cardiomyocytes, is circadian clock dependent. This diurnal variation of the KChIP2 expression is thought to increase vulnerability to ventricular arrhythmias (Jeyaraj et al., 2012).

Extended “7. Approaches related to environmental risk factors and “chrono” therapy for cardio-protection”

Misalignment of eating patterns occurs in patients who suffer from night eating syndrome (NES). This disorder causes delays in sleeping patterns, melatonin onset and rhythmic release of insulin and leptin. NES sufferers are more likely to be obese and are at increased risk of developing mood disorders such as depression (Stunkard, Grace & Wolff, 1955). Individuals who smoke have a decreased in blood melatonin levels (Ozguner, Koyu & Cesur, 2005), tend to have altered circadian rhythms and are at a higher risk of incident MI (Lopez Messa, Garmendia Leiza, Aguilar Garcia, Andres de Llano, Alberola Lopez & Ardura Fernandez, 2004). Chronic alcohol consumption resets the circadian clock; melatonin

levels in alcoholics show higher levels during the day than the night time (Hasler, Bootzin, Cousins, Fridel & Wenk, 2008). Adolescents who completed substance abuse programmes showed a positive association between their level of substance abuse and circadian phase (Hasler, Bootzin, Cousins, Fridel & Wenk, 2008). Similar results were found in animal studies, which are hypothesised to be a result of alcohol directly affecting the central clock (Brager, Ruby, Prosser & Glass, 2010; Ruby, Brager, DePaul, Prosser & Glass, 2009).

Diet rich in coumarins inhibits the melatonin metabolism (Wang et al., 2016), while a healthy diet such as a Mediterranean diet can increase the blood levels of melatonin and improve circadian rhythm (Jiki, Lecour & Nduhirabandi, 2018). Chronic and moderate consumption of melatonin at dietary doses reduces infarct size in rats/mice subjected to MI (Lamont, Nduhirabandi, Adam, Thomas, Opie & Lecour, 2015; Lamont, Somers, Lacerda, Opie & Lecour, 2011) and improves cardiac function in a rat model of pulmonary arterial hypertension (Maarman et al., 2015). Similarly, exercise can activate melatonin signalling (Qiu, Liu, Zhang, Wu, Xiao & Shi, 2018). The exercise time of the day may influence the saliva melatonin response and morning exercise rather than afternoon exercise to restore circadian rhythm (Carlson, Pobocik, Lawrence, Brazeau & Koch, 2019).

In preclinical studies, administering angiotensin converting enzyme inhibitors (ACEi), such as captopril, near sleep time reduced cardiac remodelling and improved pressure overload induced cardiac hypertrophy compared to administration of the drug during “day time” in mice. This mirrors the diurnal pattern of the renin-angiotensin system, which means that administering the drug at sleep time can increase the efficacy and target the angiotensin converting enzyme (ACE) pathway more effectively (Martino et al., 2011). Administering aspirin in the evening reduces platelet reactivity seen in the morning, which may reduce the overall risk of developing MI more effectively (Bonten et al., 2014).

For example, mice undergoing experimental AMI have a time dependent tolerance to ischemia/reperfusion (I/R) injury (Durgan et al., 2010). In both mice and humans, the size of the MI and cardiac function appear to be circadian dependent. Infarct size was significantly higher when the onset of ST-segment–elevation myocardial infarction (STEMI) occurred in the dark-to-morning phase (6am-

midday) of the day compared to the onset of STEMI in the light-to-dark phase (6pm-midnight) (Suarez-Barrientos et al., 2011).

Extended References

(ATSDR) AfTSDR (2015). The Priority List of Hazardous Substances That Will Be the Candidates for Toxicological Profiles. In https://wwwatsdrcdcgov/spl/.

Almenara CC, Broseghini-Filho GB, Vescovi MV, Angeli JK, Faria Tde O, Stefanon I, *et al.* (2013). Chronic cadmium treatment promotes oxidative stress and endothelial damage in isolated rat aorta. PLoS One 8**:** e68418.

Argacha JF, Mizukami T, Bourdrel T, & Bind MA (2019). Ecology of the cardiovascular system: Part II - A focus on non-air related pollutants. Trends Cardiovasc Med 29**:** 274-282.

Asher G, Gatfield D, Stratmann M, Reinke H, Dibner C, Kreppel F, *et al.* (2008). SIRT1 regulates circadian clock gene expression through PER2 deacetylation. Cell 134**:** 317-328.

Asher G, Reinke H, Altmeyer M, Gutierrez-Arcelus M, Hottiger MO, & Schibler U (2010). Poly(ADP-ribose) polymerase 1 participates in the phase entrainment of circadian clocks to feeding. Cell 142**:** 943-953.

Barchowsky A, Dudek EJ, Treadwell MD, & Wetterhahn KE (1996). Arsenic induces oxidant stress and NF-kappa B activation in cultured aortic endothelial cells. Free Radic Biol Med 21**:** 783-790.

Barregard L, Sallsten G, Fagerberg B, Borne Y, Persson M, Hedblad B, *et al.* (2016). Blood Cadmium Levels and Incident Cardiovascular Events during Follow-up in a Population-Based Cohort of Swedish Adults: The Malmo Diet and Cancer Study. Environmental health perspectives 124**:** 594-600.

Bartlang MS, Neumann ID, Slattery DA, Uschold-Schmidt N, Kraus D, Helfrich-Forster C, *et al.* (2012). Time matters: pathological effects of repeated psychosocial stress during the active, but not inactive, phase of male mice. The Journal of endocrinology 215**:** 425-437.

Bartlang MS, Oster H, & Helfrich-Forster C (2015). Repeated psychosocial stress at night affects the circadian activity rhythm of male mice. J Biol Rhythms 30**:** 228-241.

Bartlang MS, Savelyev SA, Johansson AS, Reber SO, Helfrich-Forster C, & Lundkvist GB (2014). Repeated psychosocial stress at night, but not day, affects the central molecular clock. Chronobiol Int 31**:** 996-1007.

Beelen R, Stafoggia M, Raaschou-Nielsen O, Andersen ZJ, Xun WW, Katsouyanni K, *et al.* (2014). Long-term exposure to air pollution and cardiovascular mortality: an analysis of 22 European cohorts. Epidemiology 25**:** 368-378.

Beutel ME, Junger C, Klein EM, Wild P, Lackner K, Blettner M, *et al.* (2016). Noise Annoyance Is Associated with Depression and Anxiety in the General Population- The Contribution of Aircraft Noise. PLoS One 11**:** e0155357.

Bonten TN, Saris A, van Oostrom MJ, Snoep JD, Rosendaal FR, Zwaginga J, *et al.* (2014). Effect of aspirin intake at bedtime versus on awakening on circadian rhythm of platelet reactivity. A randomised cross-over trial. Thromb Haemost 112**:** 1209-1218.

Bradley TD, & Floras JS (2003). Sleep Apnea and Heart Failure.

Brager AJ, Ruby CL, Prosser RA, & Glass JD (2010). Chronic ethanol disrupts circadian photic entrainment and daily locomotor activity in the mouse. Alcohol Clin Exp Res 34**:** 1266-1273.

Bray MS, Shaw CA, Moore MW, Garcia RA, Zanquetta MM, Durgan DJ*, et al.* (2008). Disruption of the circadian clock within the cardiomyocyte influences myocardial contractile function, metabolism, and gene expression. Am J Physiol Heart Circ Physiol 294**:** H1036-1047.

Breton CV, Song AY, Xiao J, Kim SJ, Mehta HH, Wan J*, et al.* (2019). Effects of air pollution on mitochondrial function, mitochondrial DNA methylation, and mitochondrial peptide expression. Mitochondrion 46**:** 22-29.

Brook RD, Rajagopalan S, Pope CA, 3rd, Brook JR, Bhatnagar A, Diez-Roux AV*, et al.* (2010). Particulate matter air pollution and cardiovascular disease: An update to the scientific statement from the American Heart Association. Circulation 121**:** 2331-2378.

Calderon-Garciduenas L, Gonzalez-Maciel A, Mukherjee PS, Reynoso-Robles R, Perez-Guille B, Gayosso-Chavez C*, et al.* (2019). Combustion- and friction-derived magnetic air pollution nanoparticles in human hearts. Environmental research 176**:** 108567.

Cao F, Souders Ii CL, Perez-Rodriguez V, & Martyniuk CJ (2018). Elucidating Conserved Transcriptional Networks Underlying Pesticide Exposure and Parkinson's Disease: A Focus on Chemicals of Epidemiological Relevance. Frontiers in genetics 9**:** 701.

Carlson LA, Pobocik KM, Lawrence MA, Brazeau DA, & Koch AJ (2019). Influence of Exercise Time of Day on Salivary Melatonin Responses. Int J Sports Physiol Perform 14**:** 351-353.

Chau NP, Mallion JM, de Gaudemaris R, Ruche E, Siche JP, Pelen O*, et al.* (1989). Twenty-four-hour ambulatory blood pressure in shift workers. Circulation 80**:** 341-347.

Chaves I, van der Horst GT, Schellevis R, Nijman RM, Koerkamp MG, Holstege FC*, et al.* (2014). Insulin-FOXO3 signaling modulates circadian rhythms via regulation of clock transcription. Curr Biol 24**:** 1248-1255.

Chevalier N, & Fenichel P (2015). Endocrine disruptors: new players in the pathophysiology of type 2 diabetes? Diabetes & metabolism 41**:** 107-115.

Clark C, & Paunovic K (2018a). WHO Environmental Noise Guidelines for the European Region: A Systematic Review on Environmental Noise and Cognition. International journal of environmental research and public health 15.

Clark C, & Paunovic K (2018b). WHO Environmental Noise Guidelines for the European Region: A Systematic Review on Environmental Noise and Quality of Life, Wellbeing and Mental Health. International journal of environmental research and public health 15.

Claudel T, Cretenet G, Saumet A, & Gachon F (2007). Crosstalk between xenobiotics metabolism and circadian clock. FEBS letters 581**:** 3626-3633.

Collaborators GBDRF (2017). Global, regional, and national comparative risk assessment of 84 behavioural, environmental and occupational, and metabolic risks or clusters of risks, 1990-2016: a systematic analysis for the Global Burden of Disease Study 2016. Lancet 390**:** 1345-1422.

Collins HE, & Rodrigo GC (2010). Inotropic response of cardiac ventricular myocytes to beta-adrenergic stimulation with isoproterenol exhibits diurnal variation: involvement of nitric oxide. Circ Res 106**:** 1244-1252.

Cosselman KE, Navas-Acien A, & Kaufman JD (2015). Environmental factors in cardiovascular disease. Nat Rev Cardiol 12**:** 627-642.

Cuypers A, Plusquin M, Remans T, Jozefczak M, Keunen E, Gielen H, *et al.* (2010). Cadmium stress: an oxidative challenge. Biometals 23**:** 927-940.

Czarna A, Berndt A, Singh HR, Grudziecki A, Ladurner AG, Timinszky G, *et al.* (2013). Structures of Drosophila cryptochrome and mouse cryptochrome1 provide insight into circadian function. Cell 153**:** 1394-1405.

Daiber A, Steven S, Weber A, Shuvaev VV, Muzykantov VR, Laher I, *et al.* (2017). Targeting vascular (endothelial) dysfunction. Br J Pharmacol 174**:** 1591-1619.

DALYs GBD, & Collaborators H (2016). Global, regional, and national disability-adjusted life-years (DALYs) for 315 diseases and injuries and healthy life expectancy (HALE), 1990-2015: a systematic analysis for the Global Burden of Disease Study 2015. Lancet 388**:** 1603-1658.

Drechsel DA, & Patel M (2008). Role of reactive oxygen species in the neurotoxicity of environmental agents implicated in Parkinson's disease. Free Radic Biol Med 44**:** 1873-1886.

Durgan DJ, Pulinilkunnil T, Villegas-Montoya C, Garvey ME, Frangogiannis NG, Michael LH, *et al.* (2010). Short communication: ischemia/reperfusion tolerance is time-of-day-dependent: mediation by the cardiomyocyte circadian clock. Circ Res 106**:** 546-550.

Englund A, Behrens S, Wegscheider K, & Rowland E (1999). Circadian variation of malignant ventricular arrhythmias in patients with ischemic and nonischemic heart disease after cardioverter defibrillator implantation. European 7219 Jewel Investigators. J Am Coll Cardiol 34**:** 1560-1568.

Fransson EI, Nyberg ST, Heikkila K, Alfredsson L, Bjorner JB, Borritz M, *et al.* (2015). Job strain and the risk of stroke: an individual-participant data meta-analysis. Stroke; a journal of cerebral circulation 46**:** 557-559.

Furlan R, Barbic F, Piazza S, Tinelli M, Seghizzi P, & Malliani A (2000). Modifications of Cardiac Autonomic Profile Associated With a Shift Schedule of Work.

GBD 2016 Risk Factors Collaborators (2017). Global, regional, and national comparative risk assessment of 84 behavioural, environmental and occupational, and metabolic risks or clusters of risks, 1990–2016: a systematic analysis for the Global Burden of Disease Study 2016. Lancet 390**:** 1345-1422.

Ghezzi P, Floridi L, Boraschi D, Cuadrado A, Manda G, Levic S, *et al.* (2018). Oxidative Stress and Inflammation Induced by Environmental and Psychological Stressors: A Biomarker Perspective. Antioxid Redox Signal 28**:** 852-872.

Hahad O, Beutel M, Gori T, Schulz A, Blettner M, Pfeiffer N, *et al.* (2018). Annoyance to different noise sources is associated with atrial fibrillation in the Gutenberg Health Study. Int J Cardiol 264**:** 79-84.

Han C, & Hong YC (2016). Bisphenol A, Hypertension, and Cardiovascular Diseases: Epidemiological, Laboratory, and Clinical Trial Evidence. Current hypertension reports 18**:** 11.

Hart JE, Chiuve SE, Laden F, & Albert CM (2014). Roadway proximity and risk of sudden cardiac death in women. Circulation 130**:** 1474-1482.

Hasler BP, Bootzin RR, Cousins JC, Fridel K, & Wenk GL (2008). Circadian phase in sleep-disturbed adolescents with a history of substance abuse: a pilot study. Behav Sleep Med 6**:** 55-73.

Hickie IB, Naismith SL, Robillard R, Scott EM, & Hermens DF (2013). Manipulating the sleep-wake cycle and circadian rhythms to improve clinical management of major depression. BMC Med 11**:** 79.

Huang W, Wang G, Lu SE, Kipen H, Wang Y, Hu M*, et al.* (2012). Inflammatory and oxidative stress responses of healthy young adults to changes in air quality during the Beijing Olympics. American journal of respiratory and critical care medicine 186**:** 1150-1159.

Humblet O, Birnbaum L, Rimm E, Mittleman MA, & Hauser R (2008). Dioxins and cardiovascular disease mortality. Environmental health perspectives 116**:** 1443-1448.

Jeyaraj D, Haldar SM, Wan X, McCauley MD, Ripperger JA, Hu K*, et al.* (2012). Circadian rhythms govern cardiac repolarization and arrhythmogenesis. Nature 483**:** 96-99.

Jiki Z, Lecour S, & Nduhirabandi F (2018). Cardiovascular Benefits of Dietary Melatonin: A Myth or a Reality? Front Physiol 9.

Jordan SD, & Lamia KA (2013). AMPK at the crossroads of circadian clocks and metabolism. Molecular and cellular endocrinology 366**:** 163-169.

Kahn LG, & Trasande L (2018). Environmental Toxicant Exposure and Hypertensive Disorders of Pregnancy: Recent Findings. Current hypertension reports 20**:** 87.

Karoui A, Crochemore C, Mulder P, Preterre D, Cazier F, Dewaele D*, et al.* (2019). An integrated functional and transcriptomic analysis reveals that repeated exposure to diesel exhaust induces sustained mitochondrial and cardiac dysfunctions. Environ Pollut 246**:** 518-526.

Kempen EV, Casas M, Pershagen G, & Foraster M (2018). WHO Environmental Noise Guidelines for the European Region: A Systematic Review on Environmental Noise and Cardiovascular and Metabolic Effects: A Summary. Int J Environ Res Public Health 15.

Kipen H, Rich D, Huang W, Zhu T, Wang G, Hu M*, et al.* (2010). Measurement of inflammation and oxidative stress following drastic changes in air pollution during the Beijing Olympics: a panel study approach. Ann N Y Acad Sci 1203**:** 160-167.

Koch CE, Leinweber B, Drengberg BC, Blaum C, & Oster H (2017). Interaction between circadian rhythms and stress. Neurobiol Stress 6**:** 57-67.

Kroller-Schon S, Daiber A, Steven S, Oelze M, Frenis K, Kalinovic S*, et al.* (2018). Crucial role for Nox2 and sleep deprivation in aircraft noise-induced vascular and cerebral oxidative stress, inflammation, and gene regulation. Eur Heart J 39**:** 3528-3539.

Kumagai Y, & Pi J (2004). Molecular basis for arsenic-induced alteration in nitric oxide production and oxidative stress: implication of endothelial dysfunction. Toxicology and applied pharmacology 198**:** 450-457.

Lamia KA, Sachdeva UM, DiTacchio L, Williams EC, Alvarez JG, Egan DF*, et al.* (2009). AMPK regulates the circadian clock by cryptochrome phosphorylation and degradation. Science 326**:** 437-440.

Lamont K, Nduhirabandi F, Adam T, Thomas DP, Opie LH, & Lecour S (2015). Role of melatonin, melatonin receptors and STAT3 in the cardioprotective effect of chronic and moderate consumption of red wine. Biochem Biophys Res Commun 465**:** 719-724.

Lamont KT, Somers S, Lacerda L, Opie LH, & Lecour S (2011). Is red wine a SAFE sip away from cardioprotection? Mechanisms involved in resveratrol- and melatonin-induced cardioprotection. J Pineal Res 50**:** 374-380.

Landrigan PJ, Fuller R, Acosta NJR, Adeyi O, Arnold R, Basu NN*, et al.* (2018). The Lancet Commission on pollution and health. Lancet 391**:** 462-512.

Lee Y, & Kim EK (2013). AMP-activated protein kinase as a key molecular link between metabolism and clockwork. Experimental & molecular medicine 45**:** e33.

Leka S, Jain A, & Organization WH (2010) *Health impact of psychosocial hazards at work: an overview*. World Health Organization.

Lelieveld J, Haines A, & Pozzer A (2018). Age-dependent health risk from ambient air pollution: a modelling and data analysis of childhood mortality in middle-income and low-income countries. Lancet Planet Health 2**:** e292-e300.

Lim SS, Vos T, Flaxman AD, Danaei G, Shibuya K, Adair-Rohani H*, et al.* (2012). A comparative risk assessment of burden of disease and injury attributable to 67 risk factors and risk factor clusters in 21 regions, 1990-2010: a systematic analysis for the Global Burden of Disease Study 2010. Lancet 380**:** 2224-2260.

Lind L, & Lind PM (2012). Can persistent organic pollutants and plastic-associated chemicals cause cardiovascular disease? Journal of internal medicine 271**:** 537-553.

Lind PM, & Lind L (2018). Endocrine-disrupting chemicals and risk of diabetes: an evidence-based review. Diabetologia 61**:** 1495-1502.

Lopez Messa JB, Garmendia Leiza JR, Aguilar Garcia MD, Andres de Llano JM, Alberola Lopez C, & Ardura Fernandez J (2004). [Cardiovascular risk factors in the circadian rhythm of acute myocardial infarction]. Rev Esp Cardiol 57**:** 850-858.

Maarman G, Blackhurst D, Thienemann F, Blauwet L, Butrous G, Davies N*, et al.* (2015). Melatonin as a preventive and curative therapy against pulmonary hypertension. J Pineal Res 59**:** 343-353.

Malisch JL, Saltzman W, Gomes FR, Rezende EL, Jeske DR, & Garland T, Jr. (2007). Baseline and stress-induced plasma corticosterone concentrations of mice selectively bred for high voluntary wheel running. Physiol Biochem Zool 80**:** 146-156.

Martino TA, Tata N, Belsham DD, Chalmers J, Straume M, Lee P, *et al.* (2007). Disturbed diurnal rhythm alters gene expression and exacerbates cardiovascular disease with rescue by resynchronization. Hypertension 49**:** 1104-1113.

Martino TA, Tata N, Simpson JA, Vanderlaan R, Dawood F, Kabir MG, *et al.* (2011). The primary benefits of angiotensin-converting enzyme inhibition on cardiac remodeling occur during sleep time in murine pressure overload hypertrophy. J Am Coll Cardiol 57**:** 2020-2028.

Marvar PJ, Vinh A, Thabet S, Lob HE, Geem D, Ressler KJ, *et al.* (2012). T lymphocytes and vascular inflammation contribute to stress-dependent hypertension. Biological psychiatry 71**:** 774-782.

Masuo Y, & Ishido M (2011). Neurotoxicity of endocrine disruptors: possible involvement in brain development and neurodegeneration. J Toxicol Environ Health B Crit Rev 14**:** 346-369.

Melvin SD, Buck DR, & Fabbro LD (2016). Diurnal activity patterns as a sensitive behavioural outcome in fish: effect of short-term exposure to treated sewage and a sub-lethal PPCP mixture. J Appl Toxicol 36**:** 1173-1182.

Menke A, Muntner P, Batuman V, Silbergeld EK, & Guallar E (2006). Blood lead below 0.48 micromol/L (10 microg/dL) and mortality among US adults. Circulation 114**:** 1388-1394.

Messner B, & Bernhard D (2010). Cadmium and cardiovascular diseases: cell biology, pathophysiology, and epidemiological relevance. Biometals 23**:** 811-822.

Messner B, Knoflach M, Seubert A, Ritsch A, Pfaller K, Henderson B, *et al.* (2009). Cadmium is a novel and independent risk factor for early atherosclerosis mechanisms and in vivo relevance. Arterioscler Thromb Vasc Biol 29**:** 1392-1398.

Meyer T, & Wirtz PH (2018). Mechanisms of Mitochondrial Redox Signaling in Psychosocial Stress-Responsive Systems: New Insights into an Old Story. Antioxid Redox Signal 28**:** 760-772.

Miguel V, Cui JY, Daimiel L, Espinosa-Diez C, Fernandez-Hernando C, Kavanagh TJ, *et al.* (2018). The Role of MicroRNAs in Environmental Risk Factors, Noise-Induced Hearing Loss, and Mental Stress. Antioxid Redox Signal 28**:** 773-796.

Moon KA, Guallar E, Umans JG, Devereux RB, Best LG, Francesconi KA, *et al.* (2013). Association between exposure to low to moderate arsenic levels and incident cardiovascular disease. A prospective cohort study. Annals of internal medicine 159**:** 649-659.

Munzel T, Gori T, Al-Kindi S, Deanfield J, Lelieveld J, Daiber A, *et al.* (2018). Effects of gaseous and solid constituents of air pollution on endothelial function. Eur Heart J 39**:** 3543-3550.

Munzel T, Schmidt FP, Steven S, Herzog J, Daiber A, & Sorensen M (2018). Environmental Noise and the Cardiovascular System. J Am Coll Cardiol 71**:** 688-697.

Munzel T, Sorensen M, Gori T, Schmidt FP, Rao X, Brook FR, *et al.* (2016). Environmental stressors and cardio-metabolic disease: part II-mechanistic insights. Eur Heart J.

Murray CJ, Ezzati M, Flaxman AD, Lim S, Lozano R, Michaud C, *et al.* (2012). GBD 2010: design, definitions, and metrics. Lancet 380**:** 2063-2066.

Naito Y, Tsujino T, Kawasaki D, Okumura T, Morimoto S, Masai M, *et al.* (2003). Circadian gene expression of clock genes and plasminogen activator inhibitor-1 in heart and aorta of spontaneously hypertensive and Wistar-Kyoto rats. Journal of hypertension 21**:** 1107-1115.

Nakahata Y, Kaluzova M, Grimaldi B, Sahar S, Hirayama J, Chen D, *et al.* (2008). The NAD+-dependent deacetylase SIRT1 modulates CLOCK-mediated chromatin remodeling and circadian control. Cell 134**:** 329-340.

Navas-Acien A, Guallar E, Silbergeld EK, & Rothenberg SJ (2007). Lead exposure and cardiovascular disease--a systematic review. Environmental health perspectives 115**:** 472-482.

Navas-Acien A, Silbergeld EK, Sharrett R, Calderon-Aranda E, Selvin E, & Guallar E (2005). Metals in urine and peripheral arterial disease. Environmental health perspectives 113**:** 164-169.

Nawrot TS, Perez L, Kunzli N, Munters E, & Nemery B (2011). Public health importance of triggers of myocardial infarction: a comparative risk assessment. Lancet 377**:** 732-740.

Nawrot TS, Saenen ND, Schenk J, Janssen BG, Motta V, Tarantini L, *et al.* (2018). Placental circadian pathway methylation and in utero exposure to fine particle air pollution. Environ Int 114**:** 231-241.

Newby DE, Mannucci PM, Tell GS, Baccarelli AA, Brook RD, Donaldson K, *et al.* (2015). Expert position paper on air pollution and cardiovascular disease. Eur Heart J 36**:** 83-93b.

Nigra AE, Ruiz-Hernandez A, Redon J, Navas-Acien A, & Tellez-Plaza M (2016). Environmental Metals and Cardiovascular Disease in Adults: A Systematic Review Beyond Lead and Cadmium. Curr Environ Health Rep 3**:** 416-433.

Nino SA, Morales-Martinez A, Chi-Ahumada E, Carrizales L, Salgado-Delgado R, Perez-Severiano F, *et al.* (2019). Arsenic Exposure Contributes to the Bioenergetic Damage in an Alzheimer's Disease Model. ACS Chem Neurosci 10**:** 323-336.

Nyberg ST, Fransson EI, Heikkila K, Ahola K, Alfredsson L, Bjorner JB, *et al.* (2014). Job strain as a risk factor for type 2 diabetes: a pooled analysis of 124,808 men and women. Diabetes care 37**:** 2268-2275.

Ochiai M, Iida M, Agusa T, Takaguchi K, Fujii S, Nomiyama K, *et al.* (2018). Effects of 4-Hydroxy-2,3,3',4',5-Pentachlorobiphenyl (4-OH-CB107) on Liver Transcriptome in Rats: Implication in the Disruption of Circadian Rhythm and Fatty Acid Metabolism. Toxicological sciences : an official journal of the Society of Toxicology 165**:** 118-130.

Ozguner F, Koyu A, & Cesur G (2005). Active smoking causes oxidative stress and decreases blood melatonin levels. Toxicol Ind Health 21**:** 21-26.

Polo A, Crispo A, Cerino P, Falzone L, Candido S, Giudice A, *et al.* (2017). Environment and bladder cancer: molecular analysis by interaction networks. Oncotarget 8**:** 65240-65252.

Prozialeck WC, Edwards JR, Nebert DW, Woods JM, Barchowsky A, & Atchison WD (2008). The vascular system as a target of metal toxicity. Toxicological sciences : an official journal of the Society of Toxicology 102**:** 207-218.

Qiu F, Liu X, Zhang Y, Wu Y, Xiao D, & Shi L (2018). Aerobic exercise enhanced endothelium-dependent vasorelaxation in mesenteric arteries in spontaneously hypertensive rats: the role of melatonin. Hypertens Res 41**:** 718-729.

Rao X, Zhong J, Brook RD, & Rajagopalan S (2018). Effect of Particulate Matter Air Pollution on Cardiovascular Oxidative Stress Pathways. Antioxid Redox Signal 28**:** 797-818.

Razzoli M, Karsten C, Yoder JM, Bartolomucci A, & Engeland WC (2014). Chronic subordination stress phase advances adrenal and anterior pituitary clock gene rhythms. Am J Physiol Regul Integr Comp Physiol 307**:** R198-205.

Reinke H, & Asher G (2019). Crosstalk between metabolism and circadian clocks. Nature reviews Molecular cell biology 20**:** 227-241.

Rich DQ, Kipen HM, Huang W, Wang G, Wang Y, Zhu P*, et al.* (2012). Association between changes in air pollution levels during the Beijing Olympics and biomarkers of inflammation and thrombosis in healthy young adults. JAMA 307**:** 2068-2078.

Rodrigo GC, & Herbert KE (2018). Regulation of vascular function and blood pressure by circadian variation in redox signalling. Free Radic Biol Med 119**:** 115-120.

Romano A, De Giorgio B, Parolini M, Favero C, Possenti CD, Iodice S*, et al.* (2017). Methylation of the circadian Clock gene in the offspring of a free-living passerine bird increases with maternal and individual exposure to PM10. Environ Pollut 220**:** 29-37.

Rosengren A, Hawken S, Ounpuu S, Sliwa K, Zubaid M, Almahmeed WA*, et al.* (2004). Association of psychosocial risk factors with risk of acute myocardial infarction in 11119 cases and 13648 controls from 52 countries (the INTERHEART study): case-control study. Lancet 364**:** 953-962.

Rosenthal KL, Johnston MS, Shofer FS, & Poppenga RH (2005). Psittacine plasma concentrations of elements: daily fluctuations and clinical implications. J Vet Diagn Invest 17**:** 239-244.

Ross MK, Matthews AT, & Mangum LC (2014). Chemical Atherogenesis: Role of Endogenous and Exogenous Poisons in Disease Development. Toxics 2**:** 17-34.

Roy A, Gong J, Thomas DC, Zhang J, Kipen HM, Rich DQ*, et al.* (2014). The cardiopulmonary effects of ambient air pollution and mechanistic pathways: a comparative hierarchical pathway analysis. PLoS One 9**:** e114913.

Ruby CL, Brager AJ, DePaul MA, Prosser RA, & Glass JD (2009). Chronic ethanol attenuates circadian photic phase resetting and alters nocturnal activity patterns in the hamster. Am J Physiol Regul Integr Comp Physiol 297**:** R729-737.

Rutter J, Reick M, Wu LC, & McKnight SL (2001). Regulation of clock and NPAS2 DNA binding by the redox state of NAD cofactors. Science 293**:** 510-514.

Sanada K, Harada Y, Sakai M, Todo T, & Fukada Y (2004). Serine phosphorylation of mCRY1 and mCRY2 by mitogen-activated protein kinase. Genes Cells 9**:** 697-708.

Sanchez-Santed F, Colomina MT, & Herrero Hernandez E (2016). Organophosphate pesticide exposure and neurodegeneration. Cortex 74**:** 417-426.

Segall LA, Perrin JS, Walker CD, Stewart J, & Amir S (2006). Glucocorticoid rhythms control the rhythm of expression of the clock protein, Period2, in oval nucleus of the bed nucleus of the stria terminalis and central nucleus of the amygdala in rats. Neuroscience 140**:** 753-757.

Sinning C, Keller T, Abegunewardene N, Kreitner KF, Munzel T, & Blankenberg S (2010). Tako-Tsubo syndrome: dying of a broken heart? Clin Res Cardiol 99**:** 771-780.

Srivastava S, Vladykovskaya EN, Haberzettl P, Sithu SD, D'Souza SE, & States JC (2009). Arsenic exacerbates atherosclerotic lesion formation and inflammation in ApoE-/- mice. Toxicology and applied pharmacology 241**:** 90-100.

States JC, Srivastava S, Chen Y, & Barchowsky A (2009). Arsenic and cardiovascular disease. Toxicological sciences : an official journal of the Society of Toxicology 107**:** 312-323.

Steinbach K, Glogar D, Weber H, Joskowicz G, & Kaindl F (1982). Frequency and variability of ventricular premature contractions--the influence of heart rate and circadian rhythms. Pacing Clin Electrophysiol 5**:** 38-51.

Stohs SJ (1990). Oxidative stress induced by 2,3,7,8-tetrachlorodibenzo-p-dioxin (TCDD). Free Radic Biol Med 9**:** 79-90.

Stunkard AJ, Grace WJ, & Wolff HG (1955). The night-eating syndrome; a pattern of food intake among certain obese patients. Am J Med 19**:** 78-86.

Suarez-Barrientos A, Lopez-Romero P, Vivas D, Castro-Ferreira F, Nunez-Gil I, Franco E*, et al.* (2011). Circadian variations of infarct size in acute myocardial infarction. Heart 97**:** 970-976.

Sundar IK, Sellix MT, & Rahman I (2018). Redox regulation of circadian molecular clock in chronic airway diseases. Free Radic Biol Med 119**:** 121-128.

Taegtmeyer H (2000). Metabolism—the lost child of cardiology. Journal of the American College of Cardiology 36**:** 1386--1388.

Tellez-Plaza M, Jones MR, Dominguez-Lucas A, Guallar E, & Navas-Acien A (2013). Cadmium exposure and clinical cardiovascular disease: a systematic review. Current atherosclerosis reports 15**:** 356.

Thosar SS, Butler MP, & Shea SA (2018). Role of the circadian system in cardiovascular disease. J Clin Invest 128**:** 2157-2167.

Tsai JY, Kienesberger PC, Pulinilkunnil T, Sailors MH, Durgan DJ, Villegas-Montoya C*, et al.* (2010). Direct regulation of myocardial triglyceride metabolism by the cardiomyocyte circadian clock. J Biol Chem 285**:** 2918-2929.

Tyrrell J, Galloway TS, Abo-Zaid G, Melzer D, Depledge MH, & Osborne NJ (2013). High urinary tungsten concentration is associated with stroke in the National Health and Nutrition Examination Survey 1999-2010. PLoS One 8**:** e77546.

Vaziri ND (2008). Mechanisms of lead-induced hypertension and cardiovascular disease. Am J Physiol Heart Circ Physiol 295**:** H454-465.

Videla LA, Barros SB, & Junqueira VB (1990). Lindane-induced liver oxidative stress. Free Radic Biol Med 9**:** 169-179.

Vienneau D, Perez L, Schindler C, Lieb C, Sommer H, Probst-Hensch N, *et al.* (2015). Years of life lost and morbidity cases attributable to transportation noise and air pollution: A comparative health risk assessment for Switzerland in 2010. Int J Hyg Environ Health 218**:** 514-521.

Vienneau D, Schindler C, Perez L, Probst-Hensch N, & Roosli M (2015). The relationship between transportation noise exposure and ischemic heart disease: a meta-analysis. Environmental research 138**:** 372-380.

Villarreal-Calderon R, Franco-Lira M, González-Maciel A, Reynoso-Robles R, Harritt L, Pérez-Guillé B, *et al.* (2013). Up-Regulation of mRNA Ventricular PRNP Prion Protein Gene Expression in Air Pollution Highly Exposed Young Urbanites: Endoplasmic Reticulum Stress, Glucose Regulated Protein 78, and Nanosized Particles. International Journal of Molecular Sciences 14**:** 23471-23491.

Wang C, Huo X, Tian X, Xu M, Dong P, Luan Z, *et al.* (2016). Inhibition of melatonin metabolism in humans induced by chemical components from herbs and effective prediction of this risk using a computational model. Br J Pharmacol 173**:** 3261-3275.

Wild PS, Zeller T, Beutel M, Blettner M, Dugi KA, Lackner KJ, *et al.* (2012). [The Gutenberg Health Study]. Bundesgesundheitsblatt, Gesundheitsforschung, Gesundheitsschutz 55**:** 824-829.

Willich SN, Maclure M, Mittleman M, Arntz HR, & Muller JE (1993). Sudden cardiac death. Support for a role of triggering in causation. Circulation 87**:** 1442-1450.

Wilson SJ, Miller MR, & Newby DE (2018). Effects of Diesel Exhaust on Cardiovascular Function and Oxidative Stress. Antioxid Redox Signal 28**:** 819-836.

World Health Organization (2016a). Ambient air pollution: A global assessment of exposure and burden of disease. In World Health Organization,.

World Health Organization (2016b). Preventing disease through healthy environments: a global assessment of the burden of disease from environmental risksWorld Health Organization.

Xia N, & Li H (2018). Loneliness, Social Isolation, and Cardiovascular Health. Antioxid Redox Signal 28**:** 837-851.

Xing W, Busino L, Hinds TR, Marionni ST, Saifee NH, Bush MF, *et al.* (2013). SCF(FBXL3) ubiquitin ligase targets cryptochromes at their cofactor pocket. Nature 496**:** 64-68.

Yamashita T, Sekiguchi A, Iwasaki YK, Sagara K, Iinuma H, Hatano S, *et al.* (2003). Circadian variation of cardiac K+ channel gene expression. Circulation 107**:** 1917-1922.

Yang G, Wang Y, Zeng Y, Gao GF, Liang X, Zhou M, *et al.* (2013). Rapid health transition in China, 1990-2010: findings from the Global Burden of Disease Study 2010. Lancet 381**:** 1987-2015.

Yokoyama K, Araki S, Sato H, & Aono H (2000). Circadian rhythms of seven heavy metals in plasma, erythrocytes and urine in men: observation in metal workers. Ind Health 38**:** 205-212.

Yorifuji T, Kashima S, & Doi H (2016). Fine-particulate Air Pollution from Diesel Emission Control and Mortality Rates in Tokyo: A Quasi-experimental Study. Epidemiology 27**:** 769-778.

Young ME, Razeghi P, & Taegtmeyer H (2001). Clock genes in the heart: characterization and attenuation with hypertrophy. Circ Res 88**:** 1142-1150.

Young ME, Wilson CR, Razeghi P, Guthrie PH, & Taegtmeyer H (2002). Alterations of the circadian clock in the heart by streptozotocin-induced diabetes. J Mol Cell Cardiol 34**:** 223-231.

Zanetti F, Carpi A, Menabo R, Giorgio M, Schulz R, Valen G, *et al.* (2014). The cellular prion protein counteracts cardiac oxidative stress. Cardiovasc Res 104**:** 93-102.

Zheng X, Yang Z, Yue Z, Alvarez JD, & Sehgal A (2007). FOXO and insulin signaling regulate sensitivity of the circadian clock to oxidative stress. Proc Natl Acad Sci U S A 104**:** 15899-15904.
